# Supplementary material for: Regulation of protein secretion through chemical regulation of endoplasmic reticulum retention signal cleavage
Source: Nat Commun. 2022 Mar 14;13:1323. doi: 10.1038/s41467-022-28971-9 (PMC8904541; doi:10.1038/s41467-022-28971-9)
Supplement: Supplementary file 1 — Supplementary information [file 41467_2022_28971_MOESM1_ESM.pdf]

Supplementary Table 1: Amino acid sequences of constructs used in this study. Unless stated otherwise, all constructs contained the CMV promoter and were inserted in the pcDNA3 backbone.

**Potyviral proteases, ER-localized proteases and inducible split-proteases**		
№	Name and scheme of construct	Amino acid or nucleotide sequence and description of parts
1	TEVp<br>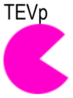	MEQKLISEEDLGESLFKGPRDYNPISSTICHLTNESDGHTTSLYGIGFGPFIITNKHLFRRNNGTLLVQSLHGVFKVKNTTTLQQHLIDGRDMI<br>IIRMPKDFPPFPQKLKFREPQREERICLVTTNFQTKSMSSMVSDTSCTFPSSDGIFWKHWIQTKDGQCGSPLVSTRDGFIVGIHSASNFTNTNN<br>YFTSVPKNFMELLTNQEAQQWVSGWRLNADSVLWGGHKVFMSKPEEPFQPVKEATQLMSELVYSQYPYDVPDYA<br><br>Dark blue: Myc tag; Magenta: TEVp; Green: HA tag
2	PPVp<br>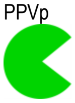	MSKSLFRGLRDYNPIASSICQLNNSSGARQSEMFGLGFGGLIVTNQHLFKRNDGELTIRSHHGEFVVKDTKTLKLLPCKGRDIVIIRLPKDFPP<br>FPKRLQFRTPTTEDRVCLIGSNFQTKSISSTMSETSATYPVDNSHFWKHWISTKDGHCGLPIVSTRDGSILGLHSLANSTNTQNFYAAFPDNFE<br>TTYLSNQDNDNWIKQWRYNPDEVCWGSLQLKRDIPQSPFTICKLLTDLDGEFVYTQ<br><br>Greem: PPVp
3	SbMVp<br>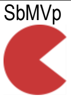	MSKSVYKGLRDYSGISTLICQLTNSSDGHKETMFGVGYGSFIITNGHLFRRNNGMLTVKTWHGEFVIHNTTQLKIHFIQGRDVILIRMPKDFPP<br>FGKRNLFRQPKREERVCMVGTNFQEKSLRATVSESSMILPEGKGSFWIHWITTQDGFCGLPLVSVNDGHIVGIHGLTSNDSEKNFFVPLTDGFE<br>KEYLENADNLSWDKHWFWEPSKIAWGSLNLVEEQPKEEFKISKLVSDLFGNTVTVQYPYDVPDYA<br><br>Dark brown: SbMVp; Dark blue: HA tag
4	SuMMVp<br>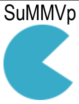	MGVSLSRGVRDYNAISSMVCRVTNDSGSSSTTMYGIGYGCYIITNKHLFRENNGRLLITSHHGEYICKNSASLKLSLVPGRDMLLIRLPKDCPP<br>FPSKLKFREPTSEEKAVLVVTNFQEKHLSSMVSESSCVVQREDSPIWRHWISTKDGHCGAPIVSIRDGYIIGSHCGENPMTSNFFTSIPKDFQN<br>LLNGKEANEWVSGWKYNIDAVCWGGLSVVNDAPSEPFITAKVVSALDTEGIKVQYPYDVPDYA<br><br>Blue: SuMMVp; Dark blue: HA tag
5	erTEVp<br>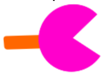	MDMRVLAQLLGLLLLCFPGARCGESLFKGPRDYNPISSTICHLTQESDGHTTSLYGIGFGPFIITNKHLFRRNNGTLLVQSLHGVFKVKNTTTL<br>QQHLIDGRDMIIIRMPKDFPPFPQKLKFREPQREERICLVTTNFQTKSMSSMVSDTSSTFPSSDGIFWKHWIQTKDGQCGSPLVSTRDGFIVGI<br>HSASNFGNTNNYFTSVPKNFMELLTNQEAQQWVSGWRLNADSVLWGGHKVFMSKPEEPFQPVKEATQLMNEGGGLEKDEL<br><br>Gray: Signaling sequence; Magenta: TEVp; Orange: KDEL signal
6	erPPVp<br>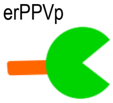	MDMRVLAQLLGLLLLCFPGARCSKSLFRGLRDYNPIASSICQLNNSSGARQSEMFGLGFGGLIVTNQHLFKRNDGELTIRSHHGEFVVKDTKTL<br>KLLPCKGRDIVIIRLPKDFPPFPKRLQFRTPTTEDRVCLIGSNFQTKSISSTMSETSATYPVDNSHFWKHWISTKDGHCGLPIVSTRDGSILGL<br>HSLANSTNTQNFYAAFPDNFETTYLSNQDNDNWIKQWRYNPDEVCWGSLQLKRDIPQSPFTICKLLTDLDGEFVYTQKDEL<br><br>Gray: Signaling sequence; Green: PPVp; Orange: KDEL signal
7	erPPVp(N23Q, T173G)<br>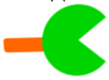	MDMRVLAQLLGLLLLCFPGARCSKSLFRGLRDYNPIASSICQLNQSSGARQSEMFGLGFGGLIVTNQHLFKRNDGELTIRSHHGEFVVKDTKTL<br>KLLPCKGRDIVIIRLPKDFPPFPKRLQFRTPTTEDRVCLIGSNFQTKSISSTMSETSATYPVDNSHFWKHWISTKDGHCGLPIVSTRDGSILGL<br>HSLANSGNTQNFYAAFPDNFETTYLSNQDNDNWIKQWRYNPDEVCWGSLQLKRDIPQSPFTICKLLTDLDGEFVYTQKDEL<br><br>Gray: Signaling sequence; Green: PPVp; Orange: KDEL signal
8	erSbMVp<br>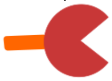	MDMRVLAQLLGLLLLCFPGARCSKSVYKGLRDYSGISTLICQLTNSSDGHKETMFGVGYGSFIITNGHLFRRNNGMLTVKTWHGEFVIHNTTQL<br>KIHFIQGRDVILIRMPKDFPPFGKRNLFRQPKREERVCMVGTNFQEKSLRATVSESSMILPEGKGSFWIHWITTQDGFCGLPLVSVNDGHIVGI<br>HGLTSNDSEKNFFVPLTDGFEKEYLENADNLSWDKHWFWEPSKIAWGSLNLVEEQPKEEFKISKLVSDLFGNTVTVQKDEL<br><br>Gray: Signaling sequence; Dark brown: SbMVp; Orange: KDEL signal
9	ABI_cTEVp<br>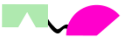	MEQKLISEEDLTRVPLYGFTSICGRRPEMEAAVSTIPRFLQSSSGSMLDGRFDPQSAAHFFGVYDGHGGSQVANYCRERMHLALAEEIAKEKPM<br>LCDGDTWLEKWKKALFNSFLRVDSEIESVAPETVGSTSVVAVVFPSHIFVANCGDSRAVLCRGKTALPLSVDHKPDREDEAARIEAAGGKVIQW<br>NGARVFGVLAMSRSIGDRYLKPSIIPDPEVTAVKRVKEDDCLILASDGVWDVMTDEEACEMARKRILLWHKKNAVAGDASLLADERRKEGKDPA<br>AMSAAEYLSKLAIQRGSKDNISVVVVDLKGSGSKSMSSMVSDTSCTFPSSDGIFWKHWIQTKDGQCGSPLVSTRDGFIVGIHSASNFTNTNNYF<br>TSVPKNFMELLTNQEAQQWVSGWRLNADSVLWGGHKVFMSKPEEPFQPVKEATQLMSELVYSQ<br><br>Dark blue: Myc tag; Black: ABI; Magenta: cTEVp
10	PYL1_nTEVp<br>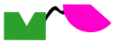	MDTYRYIGGGAPTQDEFTQLSQSIAEFHTYQLGNGRCSSLLAQRIHAPPETVWSVVRRFDRPQIYKHFIKSCNVSEDFEMRVGCTRDVNVISGL<br>PANTSRERLDLLDDDRRVTGFSITGGEHRLRNYKSVTTVHRFEKEEEEERIWTVVLESYVVDVPEGNSEEDTRLFADTVIRLNLQKLASITEAM<br>NGSGSSGESLFKGPRDYNPISSTICHLTNESDGHTTSLYGIGFGPFIITNKHLFRRNNGTLLVQSLHGVFKVKNTTTLQQHLIDGRDMIIIRMP<br>KDFPPFPQKLKFREPQREERICLVTTNFQT<br><br>Dark blue: AU1 tag; Black: PYL1; Magenta: nTEVp
11	FRB_nTEVp<br>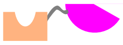	MEQKLISEEDLILWHEMWHEGLEEASRLYFGERNVKGMFEVLEPLHAMMERGPQTLKETSFNQAYGRDLMEAQEWCRKYMKSGNVKDLLQAWDL<br>YYHVFRRISKGSGSGESLFKGPRDYNPISSTICHLTNESDGHTTSLYGIGFGPFIITNKHLFRRNNGTLLVQSLHGVFKVKNTTTLQQHLIDGR<br>DMIIIRMPKDFPPFPQKLKFREPQREERICLVTTNFQT<br><br>Dark blue: Myc tag; Black: FRB; Magenta: nTEVp
11	FKBP_cTEVp<br>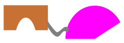	MGVQVETISPGDGRTFPKRGQTCVVHYTGMLEDGKKFDSSRDRNKPFKFMLGKQEVIRGWEEGVAQMSVGQRAKLTISPDYAYGATGHPGIIPP<br>HATLVFDVELLKLEGSGKSMSSMVSDTSCTFPSSDGIFWKHWIQTKDGQCGSPLVSTRDGFIVGIHSASNFTNTNNYFTSVPKNFMELLTNQEA<br>QQWVSGWRLNADSVLWGGHKVFMSKPEEPFQPVKEATQLMSELVYSQ<br><br>Black: FKBP; Magenta: cTEVp;
13	FRB_NerTEVp<br>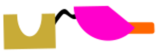	MDMRVLAQLLGLLLLCFPGARCILWHEMWHEGLEEASRLYFGERNVKGMFEVLEPLHAMMERGPQTLKETSFNQAYGRDLMEAQEWCRKYMKSG<br>NVKDLLQAWDLYYHVFRRISKGSGSGESLFKGPRDYNPISSTICHLTQESDGHTTSLYGIGFGPFIITNKHLFRRNNGTLLVQSLHGVFKVKNT<br>TTLQQHLIDGRDMIIIRMPKDFPPFPQKLKFREPQREERICLVTTNFQTKDEL<br><br>Gray: SS; Black: FRB; Magenta: nTEVp; Orange: KDEL signal
14	FKBP_CerTEVp	MDMRVLAQLLGLLLLCFPGARCGVQVETISPGDGRTFPKRGQTCVVHYTGMLEDGKKFDSSRDRNKPFKFMLGKQEVIRGWEEGVAQMSVGQRA<br>KLTISPDYAYGATGHPGIIPPHATLVFDVELLKLEGSGKSMSSMVSDTSSTFPSSDGIFWKHWIQTKDGQCGSPLVSTRDGFIVGIHSASNFGN<br>TNNYFTSVPKNFMELLTNQEAQQWVSGWRLNADSVLWGGHKVFMSKPEEPFQPVKEATQLMNEGGGLEKDEL

	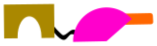	Gray: SS;Black: FKBP; Magenta: cTEVp; Orange: KDEL signal
15	ABI-CerTEVp<br>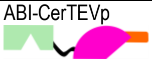	MDMRVLAQLLGLLLLCFPGARCLTRVPLYGFTSICGRRPEMEAAVSTIPRFLQSSSGSMLDGRFDPQSAAHFFGVYDGHGGSQVANYCRERMHL<br>ALAEEIAKEKPMLCDGDTWLEKWKKALFNSFLRVDSEIESVAPETVGSTSVVAVVFPSHIFVANCGDSRAVLCRGKTALPLSVDHKPDREDEAA<br>RIEAAGGKVIQWNGARVFGVLAMSRSIGDRYLKPSIIPDPEVTAVKRVKEDDCLILASDGVWDVMTDEEACEMARKRILLWHKKNAVAGDASLL<br>ADERRKEGKDPAAMSAAEYLSKLAIQRGSKDNISVVVVDLKGSGKSMSSMVSDTSSTFPSSDGIFWKHWIQTKDGQCGSPLVSTRDGFIVGIHS<br>ASNFGNTNNYFTSVPKNFMELLTNQEAQQWVSGWRLNADSVLWGGHKVFMSKPEEPFQPVKEATQLMNEGGGLEKDEL<br><br>Gray: SS; Black: ABI; Magenta: cTEVp Orange: KDEL signal
16	PYL1-NerTEVp<br>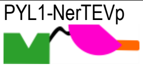	MDMRVLAQLLGLLLLCFPGARCGGGAPTQDEFTQLSQSIAEFHTYQLGNGRCSSLLAQRIHAPPETVWSVVRRFDRPQIYKHFIKSCNVSEDFE<br>MRVGCTRDVNVISGLPANTSRERLDLLDDDRRVTGFSITGGEHRLRNYKSVTTVHRFEKEEEEERIWTVVLESYVVDVPEGNSEEDTRLFADTV<br>IRLNLQKLASITEAMNGSGSGESLFKGPRDYNPISSTICHLTQESDGHTTSLYGIGFGPFIITNKHLFRRNNGTLLVQSLHGVFKVKNTTTLQQ<br>HLIDGRDMIIIRMPKDFPPFPQKLKFREPQREERICLVTTNFQTKDEL<br><br>Gray: SS; Black: PYL1; Magenta: nTEVp; Orange: KDEL signal
17	ABI-cPPVp<br>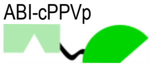	MEQKLISEEDLTRVPLYGFTSICGRRPEMEAAVSTIPRFLQSSSGSMLDGRFDPQSAAHFFGVYDGHGGSQVANYCRERMHLALAEEIAKEKPM<br>LCDGDTWLEKWKKALFNSFLRVDSEIESVAPETVGSTSVVAVVFPSHIFVANCGDSRAVLCRGKTALPLSVDHKPDREDEAARIEAAGGKVIQW<br>NGARVFGVLAMSRSIGDRYLKPSIIPDPEVTAVKRVKEDDCLILASDGVWDVMTDEEACEMARKRILLWHKKNAVAGDASLLADERRKEGKDPA<br>AMSAAEYLSKLAIQRGSKDNISVVVVDLKGSGSKSISSTMSETSATYPVDNSHFWKHWISTKDGHCGLPIVSTRDGSILGLHSLANSTNTQNFY<br>AAFPDNFETTYLSNQDNDNWIKQWRYNPDEVCWGSLQLKRDIPQSPFTICKLLTDLDGEFVYTQ<br><br>Dark blue: Myc tag; Black: ABI; Green: cPPVp
18	PYL-nPPVp<br>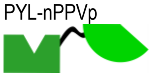	MDTYRYIGGGAPTQDEFTQLSQSIAEFHTYQLGNGRCSSLLAQRIHAPPETVWSVVRRFDRPQIYKHFIKSCNVSEDFEMRVGCTRDVNVISGL<br>PANTSRERLDLLDDDRRVTGFSITGGEHRLRNYKSVTTVHRFEKEEEEERIWTVVLESYVVDVPEGNSEEDTRLFADTVIRLNLQKLASITEAM<br>NGSGSSKSLFRGLRDYNPIASSICQLNNSSGARQSEMFGLGFGGLIVTNQHLFKRNDGELTIRSHHGEFVVKDTKTLKLLPCKGRDIVIIRLPK<br>DFPPFPKRLQFRTPTTEDRVCLIGSNFQT<br><br>Dark blue: AU1 tag; Black: PYL1; Green: nPPVp
19	FKBP_PPVs_cTEVp<br>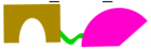	MGVQVETISPGDGRTFPKRGQTCVVHYTGMLEDGKKFDSSRDRNKPFKFMLGKQEVIRGWEEGVAQMSVGQRAKLTISPDYAYGATGHPGIIPP<br>HATLVFDVELLKLEGSGNVVVHQAGSGKSMSSMVSDTSCTFPSSDGIFWKHWIQTKDGQCGSPLVSTRDGFIVGIHSASNFTNTNNYFTSVPKN<br>FMELLTNQEAQQWVSGWRLNADSVLWGGHKVFMSKPEEPFQPVKEATQLMSELVYSQ<br><br>Black: FKBP; Green: PPVs; Magenta: cTEVp;
20	FRB_PPVs-nTEVp<br>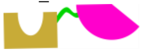	MEQKLISEEDLILWHEMWHEGLEEASRLYFGERNVKGMFEVLEPLHAMMERGPQTLKETSFNQAYGRDLMEAQEWCRKYMKSGNVKDLLQAWDL<br>YYHVFRRISKGSGNVVVHQAGSGGESLFKGPRDYNPISSTICHLTNESDGHTTSLYGIGFGPFIITNKHLFRRNNGTLLVQSLHGVFKVKNTTT<br>LQQHLIDGRDMIIIRMPKDFPPFPQKLKFREPQREERICLVTTNFQT<br><br>Dark blue: Myc tag; Black: FRB; Green: PPVs; Magenta: nTEVp

membER and lumER secretion constructs and reporters

№	Name and scheme of construct	Amino acid or nucleotide sequence and description of parts
21	SEAP<br>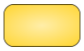	MLLLLLLGLRLQLSLGIIPVEEENPDFWNREAAEALGAAKKLQPAQTAAKNLIIFLGDGMGVSTVTAARILKGQKKDKLGPEIPLAMDRFPYV<br>ALSKTYNVDKHVPDSGATATAYLCGVKGNFQTIGLSAAARFNQCNTTRGNEVISVMNRAKKAGKSVGVVTTTRVQHASPAGTYAHTVNRNWYSD<br>ADVPASARQEGCQDIATQLISNMDIDVILGGGRKYMFRMGTPDPEYPDDYSQGGTRLDGKNLVQEWLAKRQGARYVWNRTELMQASLDPSVTHL<br>MGLFEPGDMKYEIHRDSTLDPSLMEMTEAALRLLSRNPRGFFLFVEGGRIDHGHHESRAYRALTETIMFDDAIERAGQLTSEEDTLSLVTADHS<br>HVFSFGGYPLRGSSIFGLAPGKARDRKAYTVLLYGNGPGYVLKDGARPDVTESESGSPEYRQQSAVPLDEETHAGEDVAVFARGPQAHLVHGVQ<br>EQTFIAHVMAFAACLEPYTACDLAPPAGTTDAAHPGYSRVGAAGRFEQTDTYRYIE<br><br>Gold: SEAP: Dark blue: AU1 tag;
22	SEAP_TEVs_KDEL<br>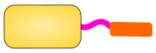	MLLLLLLGLRLQLSLGIIPVEEENPDFWNREAAEALGAAKKLQPAQTAAKNLIIFLGDGMGVSTVTAARILKGQKKDKLGPEIPLAMDRFPYV<br>ALSKTYNVDKHVPDSGATATAYLCGVKGNFQTIGLSAAARFNQCNTTRGNEVISVMNRAKKAGKSVGVVTTTRVQHASPAGTYAHTVNRNWYSD<br>ADVPASARQEGCQDIATQLISNMDIDVILGGGRKYMFRMGTPDPEYPDDYSQGGTRLDGKNLVQEWLAKRQGARYVWNRTELMQASLDPSVTHL<br>MGLFEPGDMKYEIHRDSTLDPSLMEMTEAALRLLSRNPRGFFLFVEGGRIDHGHHESRAYRALTETIMFDDAIERAGQLTSEEDTLSLVTADHS<br>HVFSFGGYPLRGSSIFGLAPGKARDRKAYTVLLYGNGPGYVLKDGARPDVTESESGSPEYRQQSAVPLDEETHAGEDVAVFARGPQAHLVHGVQ<br>EQTFIAHVMAFAACLEPYTACDLAPPAGTTDAAHPGYSRVGAAGRFEQTDTYRYIEENLYFQSKDEL<br><br>Gold: SEAP: Dark blue: AU1 tag; Black: TEV protease cleavage site; Orange: KDEL signal
23	SEAP_TM_3xTEVs_KKMP<br>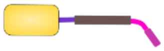	MLLLLLLGLRLQLSLGIIPVEEENPDFWNREAAEALGAAKKLQPAQTAAKNLIIFLGDGMGVSTVTAARILKGQKKDKLGPEIPLAMDRFPYV<br>ALSKTYNVDKHVPDSGATATAYLCGVKGNFQTIGLSAAARFNQCNTTRGNEVISVMNRAKKAGKSVGVVTTTRVQHASPAGTYAHTVNRNWYSD<br>ADVPASARQEGCQDIATQLISNMDIDVILGGGRKYMFRMGTPDPEYPDDYSQGGTRLDGKNLVQEWLAKRQGARYVWNRTELMQASLDPSVTHL<br>MGLFEPGDMKYEIHRDSTLDPSLMEMTEAALRLLSRNPRGFFLFVEGGRIDHGHHESRAYRALTETIMFDDAIERAGQLTSEEDTLSLVTADHS<br>HVFSFGGYPLRGSSIFGLAPGKARDRKAYTVLLYGNGPGYVLKDGARPDVTESESGSPEYRQQSAVPLDEETHAGEDVAVFARGPQAHLVHGVQ<br>EQTFIAHVMAFAACLEPYTACDLAPPAGTTDAAHPGYSRVGAAGRFEQTDTYRYIEARNRQKRGSGSARNRQKRGSGSARNRQKRSGSIIMIQT<br>LLIILFIIVPIFLLSGSGENLYFQSGSGENLYFQSGSGENLYFQSGSGKKMP<br><br>Gold: SEAP: Dark blue: AU1 tag; Furin protease cleavage site; Purple: transmembrane domain; Cyan: TEV protease cleavage site; Orange: KKMP signal
24	SEAP_PPVs_KDEL<br>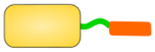	MLLLLLLGLRLQLSLGIIPVEEENPDFWNREAAEALGAAKKLQPAQTAAKNLIIFLGDGMGVSTVTAARILKGQKKDKLGPEIPLAMDRFPYV<br>ALSKTYNVDKHVPDSGATATAYLCGVKGNFQTIGLSAAARFNQCNTTRGNEVISVMNRAKKAGKSVGVVTTTRVQHASPAGTYAHTVNRNWYSD<br>ADVPASARQEGCQDIATQLISNMDIDVILGGGRKYMFRMGTPDPEYPDDYSQGGTRLDGKNLVQEWLAKRQGARYVWNRTELMQASLDPSVTHL<br>MGLFEPGDMKYEIHRDSTLDPSLMEMTEAALRLLSRNPRGFFLFVEGGRIDHGHHESRAYRALTETIMFDDAIERAGQLTSEEDTLSLVTADHS<br>HVFSFGGYPLRGSSIFGLAPGKARDRKAYTVLLYGNGPGYVLKDGARPDVTESESGSPEYRQQSAVPLDEETHAGEDVAVFARGPQAHLVHGVQ<br>EQTFIAHVMAFAACLEPYTACDLAPPAGTTDAAHPGYSRVGAAGRFEQTNVVVHQAKDEL<br><br>Gold: SEAP: Black: PPV protease cleavage site; Orange: KDEL signal
25	SEAP_SbMVs_KDEL<br>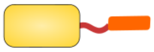	MLLLLLLGLRLQLSLGIIPVEEENPDFWNREAAEALGAAKKLQPAQTAAKNLIIFLGDGMGVSTVTAARILKGQKKDKLGPEIPLAMDRFPYV<br>ALSKTYNVDKHVPDSGATATAYLCGVKGNFQTIGLSAAARFNQCNTTRGNEVISVMNRAKKAGKSVGVVTTTRVQHASPAGTYAHTVNRNWYSD<br>ADVPASARQEGCQDIATQLISNMDIDVILGGGRKYMFRMGTPDPEYPDDYSQGGTRLDGKNLVQEWLAKRQGARYVWNRTELMQASLDPSVTHL<br>MGLFEPGDMKYEIHRDSTLDPSLMEMTEAALRLLSRNPRGFFLFVEGGRIDHGHHESRAYRALTETIMFDDAIERAGQLTSEEDTLSLVTADHS

		HVFSFGGYPLRGSSIFGLAPGKARDRKAYTVLLYGNGPGYVLKDGARPDVTESESGSPEYRQQSAVPLDEETHAGEDVAVFARGPQAHLVHGVQ<br>EQTFIAHVMAFAACLEPYTACDLAPPAGTTDAAHPGYSRVGAAGRFEQTESVSLQSKDEL<br>Gold: SEAP: Black: SbMV protease cleavage site; Orange: KDEL signal
26	SEAP_AU1_3xFURS_TM_3xTEVs_KKYL<br>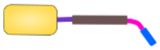	MLLLLLLLGLRLQLSLGIIPVEEENPDFWNREAAEALGAAKKLQPAQTAAKNLIIFLGDGMGVSTVTAARILKGQKKDKLGPEIPLAMDRFPYV<br>ALSKTYNVDKHVPDSGATATAYLCGVKGNFQTIGLSAAARFNQCNTTRGNEVISVMNRAKKAGKSVGVVTTTRVQHASPAGTYAHTVNRNWYSD<br>ADVPASARQEGCQDIATQLISNMDIDVILGGGRKYMFRMGTPDPEYPDDYSQGGTRLDGKNLVQEWLAKRQGARYVWNRTELMQASLDPSVTHL<br>MGLFEPGDMKYEIHRDSTLDPSLMEMTEAALRLLSRNPRGFFLFVEGGRIDHGHHESRAYRALTETIMFDDAIERAGQLTSEEDTLSLVTADHS<br>HVFSFGGYPLRGSSIFGLAPGKARDRKAYTVLLYGNGPGYVLKDGARPDVTESESGSPEYRQQSAVPLDEETHAGEDVAVFARGPQAHLVHGVQ<br>EQTFIAHVMAFAACLEPYTACDLAPPAGTTDAAHPGYSRVGAAGRFEQT<br>DTYRYIEARNRQKRGSGSARNRQKRGSGSARNRQKRSGSIIMIQTLLIILFIIVPIFLLSGSGENLYFQSGSGENLYFQSGSGENLYFQSGSGK<br>KYL<br><br>Gold: SEAP: Dark blue: AU1 tag; Furin protease cleavage site; Purple: transmembrane domain; Cyan: TEV protease cleavage site; Orange: KKYL signal
27	SEAP_AU1_3xFURS_TM_3xPPVs_KKYL<br>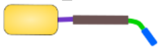	MLLLLLLLGLRLQLSLGIIPVEEENPDFWNREAAEALGAAKKLQPAQTAAKNLIIFLGDGMGVSTVTAARILKGQKKDKLGPEIPLAMDRFPYV<br>ALSKTYNVDKHVPDSGATATAYLCGVKGNFQTIGLSAAARFNQCNTTRGNEVISVMNRAKKAGKSVGVVTTTRVQHASPAGTYAHTVNRNWYSD<br>ADVPASARQEGCQDIATQLISNMDIDVILGGGRKYMFRMGTPDPEYPDDYSQGGTRLDGKNLVQEWLAKRQGARYVWNRTELMQASLDPSVTHL<br>MGLFEPGDMKYEIHRDSTLDPSLMEMTEAALRLLSRNPRGFFLFVEGGRIDHGHHESRAYRALTETIMFDDAIERAGQLTSEEDTLSLVTADHS<br>HVFSFGGYPLRGSSIFGLAPGKARDRKAYTVLLYGNGPGYVLKDGARPDVTESESGSPEYRQQSAVPLDEETHAGEDVAVFARGPQAHLVHGVQ<br>EQTFIAHVMAFAACLEPYTACDLAPPAGTTDAAHPGYSRVGAAGRFEQT<br>DTYRYIEARNRQKRGSGSARNRQKRGSGSARNRQKRSGSIIMIQTLLIILFIIVPIFLLSGSGNVVVHQAGSGNVVVHQAGSGNVVVHQAGSGK<br>KYL<br><br>Gold: SEAP; Dark blue: AU1 tag; Furin protease cleavage site; Purple: transmembrane domain; Cyan: PPV protease cleavage site; Orange: KKYL signal
28	SEAP_AU1_3xFURS_TM_3xSbMVs_KKYL<br>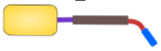	MLLLLLLLGLRLQLSLGIIPVEEENPDFWNREAAEALGAAKKLQPAQTAAKNLIIFLGDGMGVSTVTAARILKGQKKDKLGPEIPLAMDRFPYV<br>ALSKTYNVDKHVPDSGATATAYLCGVKGNFQTIGLSAAARFNQCNTTRGNEVISVMNRAKKAGKSVGVVTTTRVQHASPAGTYAHTVNRNWYSD<br>ADVPASARQEGCQDIATQLISNMDIDVILGGGRKYMFRMGTPDPEYPDDYSQGGTRLDGKNLVQEWLAKRQGARYVWNRTELMQASLDPSVTHL<br>MGLFEPGDMKYEIHRDSTLDPSLMEMTEAALRLLSRNPRGFFLFVEGGRIDHGHHESRAYRALTETIMFDDAIERAGQLTSEEDTLSLVTADHS<br>HVFSFGGYPLRGSSIFGLAPGKARDRKAYTVLLYGNGPGYVLKDGARPDVTESESGSPEYRQQSAVPLDEETHAGEDVAVFARGPQAHLVHGVQ<br>EQTFIAHVMAFAACLEPYTACDLAPPAGTTDAAHPGYSRVGAAGRFEQT<br>DTYRYIEARNRQKRGSGSARNRQKRGSGSARNRQKRSGSIIMIQTLLIILFIIVPIFLLSGSGESVSLQSGSGESVSLQSGSGESVSLQSGSGK<br>KYL<br><br>Gold: SEAP: Dark blue: AU1 tag; Furin protease cleavage site; Purple: transmembrane domain; Cyan: SbMV protease cleavage site; Orange: KKYL signal
29	SEAP_AU1_3xFURS_TM_3xSuMMVs_KKYL<br>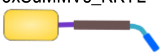	MLLLLLLLGLRLQLSLGIIPVEEENPDFWNREAAEALGAAKKLQPAQTAAKNLIIFLGDGMGVSTVTAARILKGQKKDKLGPEIPLAMDRFPYV<br>ALSKTYNVDKHVPDSGATATAYLCGVKGNFQTIGLSAAARFNQCNTTRGNEVISVMNRAKKAGKSVGVVTTTRVQHASPAGTYAHTVNRNWYSD<br>ADVPASARQEGCQDIATQLISNMDIDVILGGGRKYMFRMGTPDPEYPDDYSQGGTRLDGKNLVQEWLAKRQGARYVWNRTELMQASLDPSVTHL<br>MGLFEPGDMKYEIHRDSTLDPSLMEMTEAALRLLSRNPRGFFLFVEGGRIDHGHHESRAYRALTETIMFDDAIERAGQLTSEEDTLSLVTADHS<br>HVFSFGGYPLRGSSIFGLAPGKARDRKAYTVLLYGNGPGYVLKDGARPDVTESESGSPEYRQQSAVPLDEETHAGEDVAVFARGPQAHLVHGVQ<br>EQTFIAHVMAFAACLEPYTACDLAPPAGTTDAAHPGYSRVGAAGRFEQT<br>DTYRYIEARNRQKRGSGSARNRQKRGSGSARNRQKRSGSIIMIQTLLIILFIIVPIFLLSGSGEEIHLQSGSGEEIHLQSGSGEEIHLQSGSGK<br>KYL<br><br>Gold: SEAP: Dark blue: AU1 tag; Furin protease cleavage site; Purple: transmembrane domain; Cyan: SuMMV protease cleavage site; Orange: KKYL signal
30	Gluc-TEVs-KDEL<br>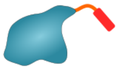	MALWMRLLPLLALLALWGPDPAAAKPTENNEDFNIVAVASNFATTDLDADRGKLPGKKLPLEVLKEMEANARKAGCTRGCLICLSHIKCTPKMK<br>KFIPGRCHTYEGDKESAQGGIGEAIVDIPEIPGFKDLEPMEQFIAQVDLCVDCTTGCLKGLANVQCSDLLKKWLPQRCATFASKIQGQVDKIKG<br>AGGDENLYFQSKDEL<br><br>Gray: Signaling sequence; Blue: Gaussia luciferase; Black: TEV protease cleavage site; Orange: KDEL signal
31	fLuc 	MEQKLISEEDLEDAKNIKKGPAPFYPLEDGTAGEQLHKAMKRYALVPGTIAFTDAHIEVDITYAEYFEMSVRLAEAMKRYGLNTNHRIVVCSEN<br>SLQFFMPVLGALFIGVAVAPANDIYNERELLNSMGISQPTVVFVSKKGLQKILNVQKKLPIIQKIIIMDSKTDYQGFQSMYTFVTSHLPPGFNE<br>YDFVPESFDRDKTIALIMNSSGSTGLPKGVALPHRTACVRFSHARDPIFGNQIIPDTAILSVVPFHHGFGMFTTLGYLICGFRVVLMYRFEEEL<br>FLRSLQDYKIQSALLVPTLFSFFAKSTLIDKYDLSNLHEIASGGAPLSKEVGEAVAKRFHLPGIRQGYGLTETTSAILITPEGDDKPGAVGKVV<br>PFFEAKVVDLDTGKTLGVNQRGELCVRGPMIMSGYVNNPEATNALIDKDGWLHSGDIAYWDEDEHFFIVDRLKSLIKYKGYQVAPAELESILLQ<br>HPNIFDAGVAGLPDDDAGELPAAVVVLEHGKSTMTEKEIVDYVASQVTTAKKLRGGVVFVDEVPKGLTGKLDARKIREILIKAKKGGKIAVNSG<br>SGYPYDVPDYA<br><br>Dark blue: Myc-tag; Black: fLuc; ; Blue: HA-tag
32	rLuc<br>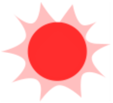	MASKVYDPEQRKRMITGPQWWARCKQMNVLDSFINYYDSEKHAENAVIFLHGNAASSYLWRHVVPHIEPVARCIIPDLIGMGKSGKSGNGSYRL<br>LDHYKYLTAWFELLNLPKKIIFVGHDWGACLAFHYSYEHQDKIKAIVHAESVVDVIESWDEWPDIEEDIALIKSEEGEKMVLENNFFVETMLPS<br>KIMRKLEPEEFAAYLEPFKEKGEVRRPTLSWPREIPLVKGGKPDVVQIVRNYNAYLRASDDLPKMFIESDPGFFSNAIVEGAKKFPNTEFVKVK<br>GLHFSQEDAPDEMGKYIKSFVERVLKNEQ<br><br>Red: *Renilla* luciferase

Constructs for inducible transcription

№	Name and scheme of construct	Amino acid or nucleotide sequence and description of parts
33	PYL1_NLS_VPR<br>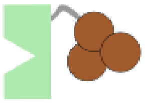	MHHHHHHGGGAPTQDEFTQLSQSIAEFHTYQLGNGRCSSLLAQRIHAPPETVWSVVRRFDRPQIYKHFIKSCNVSEDFEMRVGCTRDVNVISGL<br>PANTSRERLDLLDDDRRVTGFSITGGEHRLRNYKSVTTVHRFEKEEEEERIWTVVLESYVVDVPEGNSEEDTRLFADTVIRLNLQKLASITEAM<br>NDPKKKRKVEASGSGRADALDDFDLDMLGSDALDDFDLDMLGSDALDDFDLDMLGSDALDDFDLDMLINTSGGSGGGSGGSSQYLPDTDDRHRI<br>EEKRKRTYETFKSIMKKSPFSGPTDPRPPPRRIAVPSRSSASVPKPAPQPYPFTSSLSTINYDEFPTMVFPSGQISQASALAPAPPQVLPQAPA<br>PAPAPAMVSALAQAPAPVPVLAPGPPQAVAPPAPKPTQAGEGTLSEALLQLQFDDEDLGALLGNSTDPAVFTDLASVDNSEFQQLLNQGIPVAP<br>HTTEPMLMEYPEAITRLVTGAQRPPDPAPAPLGAPGLPNGLLSGDEDFSSIADMDFSALLGSGSGSRDSREGMFLPKPEAGSAISDVFEGREVC<br>QPKRIRPFHPPGSPWANRPLPASLAPTPTGPVHEPVGSLTPAPVPQPLDPAPAVTPEASHLLEDPDEETSQAVKALREMADTVIPQKEEAAICG<br>QMDLSHPPPRGHLDELTTTLESMTEDLNLDSPLTPELNEILDTFLNDECLLHAMHISTGLSIFDTSLF<br><br>Dark blue: His tag; Black: PYL1; Red: nuclear localization signal; Orange: transcription activation domain VPR
34	FRB_NLS_VPR	MEQKLISEEDLILWHEMWHEGLEEASRLYFGERNVKGMFEVLEPLHAMMERGPQTLKETSFNQAYGRDLMEAQEWCRKYMKSGNVKDLLQAWDL<br>YYHVFRRISKGSGSDPKKKRKVEASGSGRADALDDFDLDMLGSDALDDFDLDMLGSDALDDFDLDMLGSDALDDFDLDMLINTSGGSGGGSGGS

	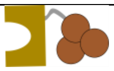	SQYLPDTDDRHRIEEKRKRTYETFKSIMKKSPFSGPTDPRPPPRRIAVPSRSSASVPKPAPQPYPFTSSLSTINYDEFPTMVFPSGQISQASAL<br>APAPPQVLPQAPAPAPAPAMVSALAQAPAPVPVLAPGPPQAVAPPAPKPTQAGEGTLSEALLQLQFDDEDLGALLGNSTDPAVFTDLASVDNSE<br>FQQLLNQGIPVAPHTTEPMLMEYPEAITRLVTGAQRPPDPAPAPLGAPGLPNGLLSGDEDFSSIADMDFSALLGSGSGSRDSREGMFLPKPEAG<br>SAISDVFEGREVCQPKRIRPFHPPGSPWANRPLPASLAPTPTGPVHEPVGSLTPAPVPQPLDPAPAVTPEASHLLEDPDEETSQAVKALREMAD<br>TVIPQKEEAAICGQMDLSHPPPRGHLDELTTTLESMTEDLNLDSPLTPELNEILDTFLNDECLLHAMHISTGLSIFDTSLF<br><br>Dark blue: Myc tag; Black: FRB; Red: nuclear localization signal; Orange: transcription activation domain VPR
35	dCas9_NLS_ABI<br>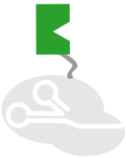	MHHHHHHDKKYSIGLAIGTNSVGWAVITDEYKVPSKKFKVLGNTDRHSIKKNLIGALLFDSGETAEATRLKRTARRRYTRRKNRICYLQEIFSN<br>EMAKVDDSFFHRLEESFLVEEDKKHERHPIFGNIVDEVAYHEKYPTIYHLRKKLVDSTDKADLRLIYLALAHMIKFRGHFLIEGDLNPDNSDVD<br>KLFIQLVQTYNQLFEENPINASGVDAKAILSARLSKSRRLENLIAQLPGEKKNGLFGNLIALSLGLTPNFKSNFDLAEDAKLQLSKDTYDDDLD<br>NLLAQIGDQYADLFLAAKNLSDAILLSDILRVNTEITKAPLSASMIKRYDEHHQDLTLLKALVRQQLPEKYKEIFFDQSKNGYAGYIDGGASQE<br>EFYKFIKPILEKMDGTEELLVKLNREDLLRKQRTFDNGSIPHQIHLGELHAILRRQEDFYPFLKDNREKIEKILTFRIPYYVGPLARGNSRFAW<br>MTRKSEETITPWNFEEVVDKGASAQSFIERMTNFDKNLPNEKVLPKHSLLYEYFTVYNELTKVKYVTEGMRKPAFLSGEQKKAIVDLLFKTNRK<br>VTVKQLKEDYFKKIECFDSVEISGVEDRFNASLGTYHDLLKIIKDKDFLDNEENEDILEDIVLTLTLFEDREMIEERLKTYAHLFDDKVMKQLK<br>RRRYTGWGRLSRKLINGIRDKQSGKTILDFLKSDGFANRNFMQLIHDDSLTFKEDIQKAQVSGQGDSLHEHIANLAGSPAIKKGILQTVKVVDE<br>LVKVMGRHKPENIVIEMARENQTTQKGQKNSRERMKRIEEGIKELGSQILKEHPVENTQLQNEKLYLYYLQNGRDMYVDQELDINRLSDYDVDA<br>IVPQSFLKDDSIDNKVLTRSDKNRGKSDNVPSEEVVKKMKNYWRQLLNAKLITQRKFDNLTKAERGGLSELDKAGFIKRQLVETRQITKHVAQI<br>LDSRMNTKYDENDKLIREVKVITLKSKLVSDFRKDFQFYKVREINNYHHAHDAYLNAVVGTALIKKYPKLESEFVYGDYKVYDVRKMIAKSEQE<br>IGKATAKYFFYSNIMNFFKTEITLANGEIRKRPLIETNGETGEIVWDKGRDFATVRKVLSMPQVNIVKKTEVQTGGFSKESILPKRNSDKLIAR<br>KKDWDPKKYGGFDSPTVAYSVLVVAKVEKGKSKKLKSVKELLGITIMERSSFEKNPIDFLEAKGYKEVKKDLIIKLPKYSLFELENGRKRMLAS<br>AGELQKGNELALPSKYVNFLYLASHYEKLKGSPEDNEQKQLFVEQHKHYLDEIIEQISEFSKRVILADANLDKVLSAYNKHRDKPIREQAENII<br>HLFTLTNLGAPAAFKYFDTTIDRKRYTSTKEVLDATLIHQSITGLYETRIDLSQLGGDDPKKKRKVTRVPLYGFTSICGRRPEMEAAVSTIPRF<br>LQSSSGSMLDGRFDPQSAAHFFGVYDGHGGSQVANYCRERMHLALAEEIAKEKPMLCDGDTWLEKWKKALFNSFLRVDSEIESVAPETVGSTSV<br>VAVVFPSHIFVANCGDSRAVLCRGKTALPLSVDHKPDREDEAARIEAAGGKVIQWNGARVFGVLAMSRSIGDRYLKPSIIPDPEVTAVKRVKED<br>DCLILASDGVWDVMTDEEACEMARKRILLWHKKNAVAGDASLLADERRKEGKDPAAMSAAEYLSKLAIQRGSKDNISVVVVDLK<br><br>Dark blue: His tag; Orange: dCas9; Red: nuclear localization signal; Black: ABI
36	dCas9_NLS_FKBP<br>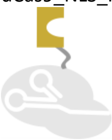	MHHHHHHDKKYSIGLAIGTNSVGWAVITDEYKVPSKKFKVLGNTDRHSIKKNLIGALLFDSGETAEATRLKRTARRRYTRRKNRICYLQEIFSN<br>EMAKVDDSFFHRLEESFLVEEDKKHERHPIFGNIVDEVAYHEKYPTIYHLRKKLVDSTDKADLRLIYLALAHMIKFRGHFLIEGDLNPDNSDVD<br>KLFIQLVQTYNQLFEENPINASGVDAKAILSARLSKSRRLENLIAQLPGEKKNGLFGNLIALSLGLTPNFKSNFDLAEDAKLQLSKDTYDDDLD<br>NLLAQIGDQYADLFLAAKNLSDAILLSDILRVNTEITKAPLSASMIKRYDEHHQDLTLLKALVRQQLPEKYKEIFFDQSKNGYAGYIDGGASQE<br>EFYKFIKPILEKMDGTEELLVKLNREDLLRKQRTFDNGSIPHQIHLGELHAILRRQEDFYPFLKDNREKIEKILTFRIPYYVGPLARGNSRFAW<br>MTRKSEETITPWNFEEVVDKGASAQSFIERMTNFDKNLPNEKVLPKHSLLYEYFTVYNELTKVKYVTEGMRKPAFLSGEQKKAIVDLLFKTNRK<br>VTVKQLKEDYFKKIECFDSVEISGVEDRFNASLGTYHDLLKIIKDKDFLDNEENEDILEDIVLTLTLFEDREMIEERLKTYAHLFDDKVMKQLK<br>RRRYTGWGRLSRKLINGIRDKQSGKTILDFLKSDGFANRNFMQLIHDDSLTFKEDIQKAQVSGQGDSLHEHIANLAGSPAIKKGILQTVKVVDE<br>LVKVMGRHKPENIVIEMARENQTTQKGQKNSRERMKRIEEGIKELGSQILKEHPVENTQLQNEKLYLYYLQNGRDMYVDQELDINRLSDYDVDA<br>IVPQSFLKDDSIDNKVLTRSDKNRGKSDNVPSEEVVKKMKNYWRQLLNAKLITQRKFDNLTKAERGGLSELDKAGFIKRQLVETRQITKHVAQI<br>LDSRMNTKYDENDKLIREVKVITLKSKLVSDFRKDFQFYKVREINNYHHAHDAYLNAVVGTALIKKYPKLESEFVYGDYKVYDVRKMIAKSEQE<br>IGKATAKYFFYSNIMNFFKTEITLANGEIRKRPLIETNGETGEIVWDKGRDFATVRKVLSMPQVNIVKKTEVQTGGFSKESILPKRNSDKLIAR<br>KKDWDPKKYGGFDSPTVAYSVLVVAKVEKGKSKKLKSVKELLGITIMERSSFEKNPIDFLEAKGYKEVKKDLIIKLPKYSLFELENGRKRMLAS<br>AGELQKGNELALPSKYVNFLYLASHYEKLKGSPEDNEQKQLFVEQHKHYLDEIIEQISEFSKRVILADANLDKVLSAYNKHRDKPIREQAENII<br>HLFTLTNLGAPAAFKYFDTTIDRKRYTSTKEVLDATLIHQSITGLYETRIDLSQLGGDDPKKKRKVGVQVETISPGDGRTFPKRGQTCVVHYTG<br>MLEDGKKFDSSRDRNKPFKFMLGKQEVIRGWEEGVAQMSVGQRAKLTISPDYAYGATGHPGIIPPHATLVFDVELLKLE<br><br>Dark blue: His tag; Orange: dCas9; Red: nuclear localization signal; Black: FKBP
37	sgRNA [ab nt]<br>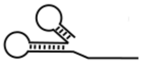	AGATGTGGAAACGGAAGAAGCGTTTTAGAGCTAGAAATAGCAAGTTAAAATAAGGCTAGTCCGTTATCAACTTGAAAAAGTGGCACCGAGTCGG<br>TGCTTTTTT<br><br>Orange: sgRNA targeting the nontemplate strand of sequence [ab]; Black: sgRNA scaffold
38	sgRNA [b]<br>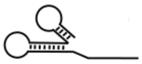	TCTTCCGTTTCCACATCTTCAGTTTTAGAGCTAGAAATAGCAAGTTAAAATAAGGCTAGTCCGTTATCAACTTGAAAAAGTGGCACCGAGTCGG<br>TGCTTTTTT<br><br>Orange: sgRNA targeting sequence [b]; Black: sgRNA scaffold

Constructs for microscopy

№	Name and scheme of construct	Amino acid or nucleotide sequence and description of parts
39	TagRFP_FURS_TM_3xTEVs_KKYL<br>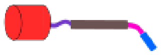	MDMRVLAQLLGLLLLCFPGARCVSKGEELIKENMHMKLYMEGTVNNHHFKCTSEGEGKPYEGTQTMRIKVVEGGPLPFAFDILATSFMYGSRTF<br>INHTQGIPDFFKQSFPEGFTWERVTTYEDGGVLTATQDTSLQDGCLIYNVKIRGVNFPSNGPVMQKKTLGWEANTEMLYPADGGLEGRSDMALK<br>LVGGGHLICNFKTTYRSKKPAKNLKMPGVYYVDHRLERIKEADKETYVEQHEVAVARYCDLPSKLGHKLNSARNRQKRGSGSGGIIMIQTLLII<br>LFIIVPIFLLSGSGENLYFQSGSGENLYFQSGSGENLYFQSGSGKKYL<br><br>Gray: signaling sequence; Red: TagRFP; Cyan: furin protease cleavage site; Green: transmembrane domain; Magenta: TEV cleavage site; Orange: KKYL signal
40	TagRFP_FURS_TM_3xTEVs<br>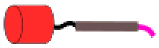	MDMRVLAQLLGLLLLCFPGARCVSKGEELIKENMHMKLYMEGTVNNHHFKCTSEGEGKPYEGTQTMRIKVVEGGPLPFAFDILATSFMYGSRTF<br>INHTQGIPDFFKQSFPEGFTWERVTTYEDGGVLTATQDTSLQDGCLIYNVKIRGVNFPSNGPVMQKKTLGWEANTEMLYPADGGLEGRSDMALK<br>LVGGGHLICNFKTTYRSKKPAKNLKMPGVYYVDHRLERIKEADKETYVEQHEVAVARYCDLPSKLGHKLNSARNRQKRGSGSGGIIMIQTLLII<br>LFIIVPIFLLSGSGENLYFQSGSGENLYFQSGSGENLYFQSGSG<br><br>Gray: signaling sequence; Red: TagRFP; Cyan: furin protease cleavage site; Green: transmembrane domain; Magenta: TEV cleavage site
41	TagRFP_AU1_3xFURS_TM_3xTEVs_KKMP<br>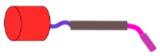	MDMRVLAQLLGLLLLCFPGARCVSKGEELIKENMHMKLYMEGTVNNHHFKCTSEGEGKPYEGTQTMRIKVVEGGPLPFAFDILATSFMYGSRTF<br>INHTQGIPDFFKQSFPEGFTWERVTTYEDGGVLTATQDTSLQDGCLIYNVKIRGVNFPSNGPVMQKKTLGWEANTEMLYPADGGLEGRSDMALK<br>LVGGGHLICNFKTTYRSKKPAKNLKMPGVYYVDHRLERIKEADKETYVEQHEVAVARYCDLPSKLGHKLNDTYRYIESARNRQKRGSGSGGIIM<br>IQTLLIIILFIIVPIFLLSGSGENLYFQSGSGENLYFQSGSGENLYFQSGSGKKMP

		Gray: signaling sequence; Red: TagRFP; Cyan: furin protease cleavage site; Green: transmembrane domain; Magenta: TEV cleavage site; Orange: KKMP signal
42	Ss_TagRFP_TEVS_KDEL<br>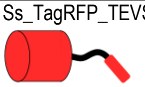	MDMRVLAQLLGLLLLCFPGARCVSKGEELIKENMHMKLYMEGTVNNHHFKCTSEGEGKPYEGTQTMRIKVVEGGPLPFAFDILATSFMYGSRTF<br>INHTQGIPDFFKQSFPEGFTWERVTTYEDGGVLTATQDTSLQDGCLIYNVKIRGVNFPSNGPVMQKKTLGWEANTEMLYPADGGLEGRSDMALK<br>LVGGGHLICNFKTTYRSKKPAKNLKMPGVYYVDHRLERIKEADKETYVEQHEVAVARYCDLPSKLGHKLNDTYRYIEENLYFQSKDEL<br><br>Black: signal sequence; Red:TagRFP; Purple:AU1 tag; Magenta: TEV protease cleavage site; Orange: KDEL signal
43	TagRFP_AU1_TM_3xTEVs<br>_KKYL<br>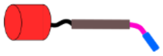	MDMRVLAQLLGLLLLCFPGARCVSKGEELIKENMHMKLYMEGTVNNHHFKCTSEGEGKPYEGTQTMRIKVVEGGPLPFAFDILATSFMYGSRTF<br>INHTQGIPDFFKQSFPEGFTWERVTTYEDGGVLTATQDTSLQDGCLIYNVKIRGVNFPSNGPVMQKKTLGWEANTEMLYPADGGLEGRSDMALK<br>LVGGGHLICNFKTTYRSKKPAKNLKMPGVYYVDHRLERIKEADKETYVEQHEVAVARYCDLPSKLGHKLNIIMIQTLLIILFIIVPIFLLSGSG<br>ENLYFQSGSGENLYFQSGSGENLYFQSGSGKKYL<br><br>Gray: signaling sequence; Red: TagRFP; Dark blue: AU1 tag; Green: transmembrane domain; Magenta: TEV cleavage site; Orange: KKYL signal
44	GFP1-10_TEVs_KDEL<br>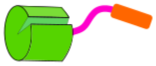	MDMRVLAQLLGLLLLCFPGARCSKGEELFTGVVPILVELDGDVNGHKFSVRGEGEGDATIGKLTLKFICTTGKLPVPWPTLVTTLTYGVQCFSR<br>YPDHMKRHDFFKSAMPEGYVQERTISFKDDGKYKTRAVVKFEGDTLVNRIELKGTDFKEDGNILGHKLEYNFNSHNVYITADKQKNGIKANFTV<br>RHNVEDGSVQLADHYQQNTPIGDGPVLLPDNHYLSTQTVLSKDPNEKDTYRYIENLYFQSKDEL<br><br>Gray: signaling sequence; Green: GFP1-10; Dark blue: AU1; Black: TEV protease cleavage site; Orange: KDEL signal
45	GFP1-<br>10_3xFURs_TM_3xTEVs_<br>KKYL<br>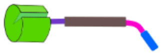	MDMRVLAQLLGLLLLCFPGARCSKGEELFTGVVPILVELDGDVNGHKFSVRGEGEGDATIGKLTLKFICTTGKLPVPWPTLVTTLTYGVQCFSR<br>YPDHMKRHDFFKSAMPEGYVQERTISFKDDGKYKTRAVVKFEGDTLVNRIELKGTDFKEDGNILGHKLEYNFNSHNVYITADKQKNGIKANFTV<br>RHNVEDGSVQLADHYQQNTPIGDGPVLLPDNHYLSTQTVLSKDPNEKARNRQKRGSGSARNRQKRGSGSARNRQKRSGSIIMIQTLLIILFIIV<br>PIFLLSGSGENLYFQSGSGENLYFQSGSGENLYFQSGSGKKYL<br><br>Gray: signaling sequence; Light green: GFP1-10; Dark green: FAS cleavage site; Purple: transmembrane domain; Cyan: TEV protease cleavage site;<br>Orange: KKYL signal
46	SS_GFP1-10_GS10_FASR<br>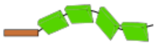	MDMRVLAQLLGLLLLCFPGARCSKGEELFTGVVPILVELDGDVNGHKFSVRGEGEGDATIGKLTLKFICTTGKLPVPWPTLVTTLTYGVQCFSR<br>YPDHMKRHDFFKSAMPEGYVQERTISFKDDGKYKTRAVVKFEGDTLVNRIELKGTDFKEDGNILGHKLEYNFNSHNVYITADKQKNGIKANFTV<br>RHNVEDGSVQLADHYQQNTPIGDGPVLLPDNHYLSTQTVLSKDPNEKGGSGGGSGGSQVTDINSKGLELRKTVTTVETQNLEGLHHDGQFCHKP<br>CPPGERKARDCTVNGDEPDCVPCQEGKEYTDKAHFSSKCRRCRLCDEGHGLEVEINCTRTQNTKCRCKPNFFCNSTVCEHCDPCTKCEHGIIKE<br>CTLTSNTKCKEEGSRSNLGWLCLLLLPIPLIVWVKRKEVQKTCRKHRKENQGSHESPTLNPETVAINL<br><br>Gray: signaling sequence; Light green: GFP1-10; Orange: Fas receptor
47	iRFP<br>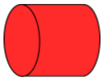	MARKVDLTSCDREPIHIPGSIQPCGCLLACDAQAVRITRITENAGAFFGRETPRVGELLADYFGETEAHALRNALAQSSDPKRPALIFGWRDGL<br>TGRTFDISLHRHDGTSIIEFEPAAAEQADNPLRLTRQIIARTKELKSLEEMAARVPRYLQAMLGYHRVMLYRFADDGSGMVIGEAKRSDLESFL<br>GQHFPASLVPQQARLLYLKNAIRVVSDSRGISSRIVPEHDASGAALDLSFAHLRSISPCHLEFLRNMGVSASMSLSIIIDGTLWGLIICHHYEP<br>RAVPMAQRVAAEMFADFLSLHFTAAHHQR<br><br>Magenta: iRFP
48	BFP<br>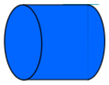	MSELIKENMHMKLYMEGTVDNHHFKCTSEGEGKPYEGTQTMRIKVVEGGPLPFAFDILATSFLYGSKTFINHTQGIPDFFKQSFPEGFTWERVT<br>TYEDGGVLTATQDTSLQDGCLIYNVKIRGVNFTSNGPVMQKKTLGWEAFTETLYPADGGLEGRNDMALKLVGGSHLIANIKTTYRSKKPAKNLK<br>MPGVYYVDYRLERIKEANNETYVEQHEVAVARYCDLPSKLGHKLN<br><br>Black: BFP
49	Fas-EnvZ(C)-mCit<br>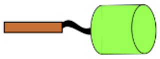	MLGIWTLLPLVLTSVARLSSKSVNAYPYDVPDYAQVTDINSKGLELRKTVTTVETQNLEGLHHDGQFCHKPCPPGERKARDCTVNGDEPDCVPC<br>QEGKEYTDKAHFSSKCRRCRLCDEGHGLEVEINCTRTQNTKCRCKPNFFCNSTVCEHCDPCTKCEHGIIKECTLTSNTKCKEEGSRSNLGWLCL<br>LLLPIPLIVWVKRKEVQKTCRKHRKENQGSHESPTLNPETVAINLGGSGGSGRIQNRPLVDLEHAALQVGKGIIPPPLREYGASEVRSVTRAFN<br>HMAAGVKQLADDRTLLMAGVSHDLRTPLTRIRLATEMMSEQDGYLAESINKDIEECNAIIEQFIDYLRTGQEMPMEMADLNAVLGEVIAAESGY<br>EREIETALYPGSIEVKMHPLSIKRAVANMVVNAARYGNGWIKVSSGTEPNRAWFQVEDDGPGIAPEQRKHLFQPFVRGDSARTISGTGLGLAIV<br>QRIVDNHNGMLELGTSERGGLSIRAWLPVPVTRAQGTTKEGGGSGVSKGEELFTGVVPILVELDGDVNGHKFSVSGEGEGDATYGKLTLKFICT<br>TGKLPVPWPTLVTTFGYGLMCFARYPDHMKQHDFFKSAMPEGYVQERTIFFKDDGNYKTRAEVKFEGDTLVNRIELKGIDFKEDGNILGHKLEY<br>NYNSHNVYIMADKQKNGIKVNFKIRHNIEDGSVQLADHYQQNTPIGDGPVLLPDNHYLSYQSALSKDPNEKRDHMVLLEFVTAAGITLGMDELY<br>KDTYRYI<br><br>Brown: Fas receptor; Orange: EnvZ(C); Light green: mCitrine; Purple AU1 tag
50	BFP-KRΦ	MEQKLISEEDLSELIKENMHMKLYMEGTVDNHHFKCTSEGEGKPYEGTQTMRIKVVEGGPLPFAFDILATSFLYGSKTFINHTQGIPDFFKQSF<br>PEGFTWERVTTYEDGGVLTATQDTSLQDGCLIYNVKIRGVNFTSNGPVMQKKTLGWEAFTETLYPADGGLEGRNDMALKLVGGSHLIANIKTTY<br>RSKKPAKNLKMPGVYYVDYRLERIKEANNETYVEQHEVAVARYCDLPSKLGHKLNDPKKKRKVGGSGGGSGGSGKKFWKRLRKFLRKLKS<br>Blue: Myc tag; black: BFP; dark green: nuclear localization signal; red: GS10 linker; purple:<br>KRΦ peptid
**Constructs for input signal processing**		
№	Name and scheme of construct	Amino acid or nucleotide sequence and description of parts
51	cTEV*-AP4-SbMVs-P3-<br>nTEV<br>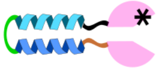	MKSMSSMVSDTSCTFPSSDGIFWKHWIQTKDGQAGSPLVSTRDGFIVGIHSASNFTNTNNYFTSVPKNFMELLTNQEAQQWVSGWRLNADSVLW<br>GGHKVFMSKPEEPFQPVKEATQLMSELVYSQYPYDVPDYAGGSGGGSGGSSPEDELAANEEELQQNEQKLAQIKQKLQAIKYGGGSGGGESVSL<br>QSGSSGSGSPEDEIQQLEEEIAQLEQKNAALKEKNQALKYGEQKLISEEDLGESLFKGPRDYNPISSTICHLTNESDGHTTSLYGIGFGPFIIT<br>NKHLFRRNNGTLLVQSLHGVFKVKNTTTLQQHLIDGRDMIIIRMPKDFPPFPQKLKFREPQREERICLVTTNFQT<br><br>Magenta: cTEVp*; Green: AP4; Red: SbMVs cleavage site; Green: P3; Dark blue: Myc tag; Magenta: nTEVp
52	P4-PPVs-cTEV<br>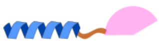	MSPEDKIAQLKQKIQALKQENQQLEEENAALEYGGGSGGGNVVVHQAKSMSSMVSDTSCTFPSSDGIFWKHWIQTKDGQCGSPLVSTRDGFIVG<br>IHSASNFTNTNNYFTSVPKNFMELLTNQEAQQWVSGWRLNADSVLWGGHKVFMSKPEEPFQPVKEATQLMSELVYSQYPYDVPDYA<br><br>Green: P4; Cyan: PPVs cleavage site; Magenta: cTEVp; Dark blue: HA tag
53	SEAP-P4<br>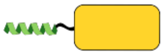	MLLLLLLLGLRLQLSLGIIPVEEENPDFWNREAAEALGAAKKLQPAQTAAKNLIIFLGDGMGVSTVTAARILKGQKKDKLGPEIPLAMDRFPYV<br>ALSKTYNVDKHVPDSGATATAYLCGVKGNFQTIGLSAAARFNQCNTTRGNEVISVMNRAKKAGKSVGVVTTTRVQHASPAGTYAHTVNRNWYSD<br>ADVPASARQEGCQDIATQLISNMDIDVILGGGRKYMFRMGTPDPEYPDDYSQGGTRLDGKNLVQEWLAKRQGARYVWNRTELMQASLDPSVTHL<br>MGLFEPGDMKYEIHRDSTLDPSLMEMTEAALRLLSRNPRGFFLFVEGGRIDHGHHESRAYRALTETIMFDDAIERAGQLTSEEDTLSLVTADHS<br>HVFSFGGYPLRGSSIFGLAPGKARDRKAYTVLLYGNGPGYVLKDGARPDVTESESGSPEYRQQSAVPLDEETHAGEDVAVFARGPQAHLVHGVQ<br>EQTFIAHVMAFAACLEPYTACDLAPPAGTTDAAHPGYSRVGAAGRFEQT<br>SGSGSPEDKIAQLKQKIQALKQENQQLEEENAALEYG<br><br>Magenta: SEAP; Green: P4 peptid

54	SEAP-P4-SbMVs-KDEL 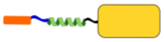	MLLLLLLLGLRLQLSLGIIPVEEENPDFWNREAAEALGAAKKLQPAQTAAKNLIIFLGDGMGVSTVTAARILKGQKKDKLGPEIPLAMDRFPYVALSKTYNVDKHVPDSGATATAYLCGVKGNFQTIGLSAAARFNQCNTTRGNEVISVMNRAKKAGKSVGVVTTTRVQHASPAGTYAHTVNRNWYSDADVPASARQEGCQDIATQLISNMDIDVILGGGRKYMFRMGTPDPEYPDDYSQGGTRLDGKNLVQEWLAKRQGARYVWNRTELMQASLDPSVTHLMGLFEPGDMKYEIHRDSTLDPSLMEMTEAALRLLSRNPRGFFLFVEGGRIDHGHHESRAYRALTETIMFDDAIERAGQLTSEEDTLSLVTADHSHVFSFGGYPLRGSSIFGLAPGKARDRKAYTVLLYGNGPGYVLKDGARPDVTESESGSPEYRQQSAVPLDEETHAGEDVAVFARGPQAHLVHGVQEQTFIAHVMAFAACLEPYTACDLAPPAGTTDAAHPGYSRVGAAGRFEQTSGSGSPEDKIAQLKQKIQALKQENQQLEEENAALEYGSGSGESVSLQSKDEL<br><br>Magenta: SEAP; Green: P4 peptid; Black: SbMV protease cleavage site; Orange: KDEL signal
55	SS-P3-TEVs-KDEL 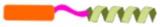	M DMRVLAQLLGLLLLCFPGARC SPEDEIQQLEEEIAQLEQKNAALKEKNQALKYGS GSG ENLYFQS KDEL<br><br>Gray: Signaling sequence; Blue: P3 peptide; Black: TEV protease cleavage site; Orange: KDEL signal

Insulin and IL10 secretion

№	Name and scheme of construct	Amino acid or nucleotide sequence and description of parts
56	Preproinsulin_TEVs-KDEL (lumER-ins.) 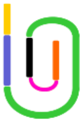	MALWMRLLPLLALLALWGPDPAAAFVNQHLCGSHLVEALYLVCGERGFFYTPRTKREAEDLQVGQVELGGGPGAGSLQPLALEGSLRQKRGIVEQCCTSICSLYQLENYCNENLYFQSKDEL<br><br>Gray: Signalling sequence; Cyan: Furin protease cleavage site; Green: C peptide Blue: B chain; Red: A chain; Black: TEV protease cleavage site; Orange: KDEL signal
57	Bchain_FURs_Gluc_FURS_Achain_TEVs_KDEL 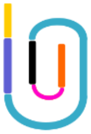	MALWMRLLPLLALLALWGPDPAAAFVNQHLCGSHLVEALYLVCGERGFFYTPRTKRKPTENNEDFNIVAVASNFATTDLDADRGKLPGKKLPLEVLKEMEANARKAGCTRGCLICLSHIKCTPKMKKFIPGRCHTYEGDKESAQGGIGEAIVDIPEIPGFKDLEPMEQFIAQVDLCVDCTTGCLKGLANVQCSDLLKKWLPQRCATFASKIQGQVDKIKGAGGDRQKRGIVEQCCTSICSLYQLENYCNENLYFQSKDEL<br><br>Gray: Signalling sequence; Cyan: Furin protease cleavage site; Green: Gaussia luciferase; Blue: B chain; Red: A chain; Black: TEV protease cleavage site; Orange: KDEL signal
58	Preproinsulin-FURS-TM-3xTEVs-KKYL (membER-ins.)	MALWMRLLPLLALLALWGPDPAAAFVNQHLCGSHLVEALYLVCGERGFFYTPRTKRKPTENNEDFNIVAVASNFATTDLDADRGKLPGKKLPLEVLKEMEANARKAGCTRGCLICLSHIKCTPKMKKFIPGRCHTYEGDKESAQGGIGEAIVDIPEIPGFKDLEPMEQFIAQVDLCVDCTTGCLKGLANVQCSDLLKKWLPQRCATFASKIQGQVDKIKGAGGDRQKRGIVEQCCTSICSLYQLENYCNARNRQKRGSGSARNRQKRGSGSARNRQKRSGSIIMIQTLLIIILFIIVPIFLLSGSGENLYFQSGSGENLYFQSGSGENLYFQSGSGKKYL<br><br>Gray: Signalling sequence; Cyan: Furin protease cleavage site; Green: Gaussia luciferase; Blue: B chain; Red: A chain; Furin protease cleavage site; Purple: transmembrane domain; Cyan: TEV protease cleavage site; Orange: KKYL signal
59	Preproinsulin 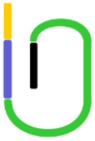	MALWMRLLPLLALLALWGPDPAAAFVNQHLCGSHLVEALYLVCGERGFFYTPRTKREAEDLQVGQVELGGGPGAGSLQPLALEGSLRQKRGIVEQCCTSICSLYQLENYCN<br><br>Gray: Signalling sequence; Cyan: Furin protease cleavage site; Green: C peptide Blue: B chain; Red: A chain
60	CAD-KKYL	MATGSRTSLLLAFGLLCLPWLQEGSAGVQVETISPGDGRTFPKRGQTCVVHYTGMLEDGKKMDSSRDRNKPFKFMLGKQEVIRGWEEGVAQMSVGQRAKLTISPDYAYGATGHPGIIPPHATLVFDVELLKLEGVQVETISPGDGRTFPKRGQTCVVHYTGMLEDGKKMDSSRDRNKPFKFMLGKQEVIRGWEEGVAQMSVGQRAKLTISPDYAYGATGHPGIIPPHATLVFDVELLKLEGVQVETISPGDGRTFPKRGQTCVVHYTGMLEDGKKMDSSRDRNKPFKFMLGKQEVIRGWEEGVAQMSVGQRAKLTISPDYAYGATGHPGIIPPHATLVFDVELLKLEGVQVETISPGDGRTFPKRGQTCVVHYTGMLEDGKKMDSSRDRNKPFKFMLGKQEVIRGWEEGVAQMSVGQRAKLTISPDYAYGATGHPGIIPPHATLVFDVELLKLESARNRQKRFVNQHLCGSHLVEALYLVCGERGFFYTPRTKREAEDLQVGQVELGGGPGAGSLQPLALEGSLRQKRGIVEQCCTSICSLYQLENYCN<br><br>Gray: signaling sequence; Blue: 4xFm [four tandem repeats of FKBP12 in which the Phe^{36} was mutated to Met] ; Black: Furin protease cleavage site; Cyan: Furin protease cleavage site; Green: C peptide Blue: B chain; Red: A chain; Black:
61	IL10-TEVs-KDEL	MHSSALLCCLVLLTGVRASPGQGTQSENSCTHFPGNLPNMLRDLRDAFSRVKTFFQMKDQLDNLLLKESLLEDFKGYLGCQALSEMIQFYLEEVMPQAENQDPDIKAHVNSLGENLKTLRLRLRRCHRFLPCENKSKAVEQVKNAFNKLQEKGIYKAMSEFDIFINYIEAYMTMKIRNENLYFQSKDEL<br><br>Blue: hIL10; Black: TEV protease cleavage site; Orange: KDEL signal

CAR-KKYL

№	Name and scheme of construct	Amino acid or nucleotide sequence and description of parts
62	ABA-TEVp(P2A) 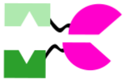	MEQKLISEEDLTRVPLYGFTSICGRRPEMEAAVSTIPRFLQSSSGSMLDGRFDPQSAAHFFGVYDGHGGSQVANYCRERMHLALAEEIAKEKPMLCDGDTWLEKWKKALFNSFLRVDSEIESVAPETVGSTSVVAVVFPSHIFVANCGDSRAVLCRGKTALPLSVDHKPDREDEAARIEAAGGKVIQWNGARVFGVLAMSRSIGDRYLKPSIIPDPEVTAVKRVKEDDCLILASDGVWDVMTDEEACEMARKRILLWHKKNAVAGDASLLADERRKEGKDPAAMSAAEYLSKLAIQRGSKDNISVVVVDLKGSGSKSMSSMVSDTSCTFPSSDGIFWKHWIQTKDGQCGSPLVSTRDGFIVGIHSASNFTNTNNYFTSVPKNFMELLTNQEAQQWVSGWRLNADSVLWGGHKVFMSKPEEPFQPVKEATQLMSELVYSQ<br>GSGATNFSLLKQAGDVEENPGP<br>MDTYRYIGGGAPTQDEFTQLSQSIAEFHTYQLGNGRCSSLLAQRIHAPPETVWSVVRRFDRPQIYKHFIKSCNVSEDFEMRVGCTRDVNVISGLPANTSRERLDLLDDDRRVTGFSITGGEHRLRNYKSVTTVHRFEKEEEEERIWTVVLESYVVDVPEGNSEEDTRLFADTVIRLNLQKLASITEAMNGSGSSGESLFKGPRDYNPISSTICHLTNESDGHTTSLYGIGFGPFIITNKHLFRRNNGTLLVQSLHGVFKVKNTTTLQQHLIDGRDMIIIRMPKDFPPFPQKLKFREPQREERICLVTTNFQT

		Dark blue: Myc tag; Black: ABI; Magenta: cTEVp; Light blue: P2A; Dark blue: AU1 tag; Black: PYL1; Magenta: nTEVp
63	CAR-KKYL<br>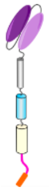	MALPVTALLLPLALLLHAARPEQKLISEEDLDIQMTQTTSSLSASLGDRVTISCRASQDISKYLNWYQQKPDGTVKLLIYHTSRLHSGVPSRFS<br>GSGSGTDYSLTISNLEQEDIATYFCQQGNTLPYTFGGGTKLEIT**GGGGSGGGGSGGGGS**EVKLQESGPGLVAPSQSLSVTCTVSGVSLPDYGVS<br>WIRQPPRKGLEWLGVIWGSETTYYNSALKSRLTIIKDNSKSQVFLKMNSLQTDDTAIYYCAKHYYYGGSYAMDYWGQGTSVTVSSTTTPAPRPP<br>TPAPTIASQPLSLRPEACRPAAGGAVHTRGLDFACDIYIWAPLAGTCGVLLLSLVITLYCKRGRKKLLYIFKQPFMRPVQTTQEEDGCSCRFPE<br>EEEGGCELRVKFSRSADAPAYQQGQNQLYNELNLGRREEYDVLDKRRGRDPEMGGKPRRKNPQEGLYNELQKDKMAEAYSEIGMKGERRRGKGH<br>DGLYQGLSTATKDTYDALHMQALPPRGSGENLYFQSGSGENLYFQSGSGENLYFQSGSGKKYL<br><br>Orange: CD8leader; Green: Myc; Light gray: Vl(CD19);**Black: (G4S)3linker**; Cyan: Vh(CD19); Purple: hinge; Red: CD8TM; Dark gray: 4-1BB; Yellow: CD3zeta; Cyan: TEV protease cleavage site; Orange: KKYL signal

FURp and KDELR1

№	Name and scheme of construct	Amino acid or nucleotide sequence and description of parts
64	Furin protease<br>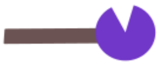	MELRPWLLWVVAATGTLVLLAADAQGQKVFTNTWAVRIPGGPAVANSVARKHGFLNLGQIFGDYYHFWHRGVTKRSLSPHRPRHSRLQREPQVQ<br>WLEQQVAKRRTKRDVYQEPTDPKFPQQWYLSGVTQRDLNVKAAWAQGYTGHGIVVSILDDGIEKNHPDLAGNYDPGASFDVNDQDPDPQPRYTQ<br>MNDNRHGTRCAGEVAAVANNGVCGVGVAYNARIGGVRMLDGEVTDAVEARSLGLNPNHIHIYSASWGPEDDGKTVDGPARLAEEAFFRGVSQGR<br>GGLGSIFVWASGNGGREHDSCNCDGYTNSIYTLSISSATQFGNVPWYSEACSSTLATTYSSGNQNEKQIVTTDLRQKCTESHTGTSASAPLAAG<br>IIALTLEANKNLTWRDMQHLVVQTSKPAHLNANDWATNGVGRKVSHSYGYGLLDAGAMVALAQNWTTVAPQRKCIIDILTEPKDIGKRLEVRKT<br>VTACLGEPNHITRLEHAQARLTLSYNRRGDLAIHLVSPMGTRSTLLAARPHDYSADGFNDWAFMTTHSWDEDPSGEWVLEIENTSEANNYGTLT<br>KFTLVLYGTAPEGLPVPPESSGCKTLTSSQACVVCEEGFSLHQKSCVQHCPPGFAPQVLDTHYSTENDVETIRASVCAPCHASCATCQGPALTD<br>CLSCPSHASLDPVEQTCSRQSQSSRESPPQQQPPRLPPEVEAGQRLRAGLLPSHLPEVVAGLSCAFIVLVFVTVFLVLQLRSGFSFRGVKVYTM<br>DRGLISYKGLPPEAWQEECPSDSEEDEGRGERTAFIKDQSAL<br><br>Magenta: furin protease
65	KDELR1<br>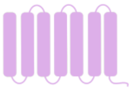	MNLFRFLGDLSHLLAIILLLLKIWKSRSCAGISGKSQVLFAVVFTARYLDLFTNYISLYNTCMKVVYIACSFTTVWLIYSKFKATYDGNHDTFR<br>VEFLVVPTAILAFLVNHDFTPLEILWTFSIYLESVAILPQLFMVSKTGEAETITSHYLFALGVYRTLYLFNWIWRYHFEGFFDLIAIVAGLVQT<br>VLYCDFFYLYITKVLKGKKLSLPA<br><br>Purple: KDELR1

Supplementary Table 2: Constructs with a minimal promoter used in this study to measure the kinetics of secretion in systems based on the induction of transcription

Plasmid name and construct illustration	Plasmid description
pcDNA 3.1/7x[b]_P_{MIN}_SEAP	Plasmid ensures expression of the SEAP reporter upon binding of a transcriptional activator to target sites upstream of a minimal promoter. The plasmid contains 7 copies of target sites [b].
pcDNA 3.1/10[ab]_P_{MIN}_preproinsulin	Plasmid ensures expression of the preproinsulin upon binding of a transcriptional activator to target sites upstream of a minimal promoter. The plasmid contains 10 copies of target sites [ab].
U6_gRNA[b]	Plasmid ensures constitutive expression of gRNA[b], driven from the murine U6 promoter. The gRNA targets a sequence designated [b].
U6_gRNA[ab nt]	Plasmid ensures constitutive expression of gRNA[ab nt], driven from the murine U6 promoter. The gRNA targets the non-template (nt) strand of a sequence designated [ab].

Supplementary Table 3: Nucleotide sequence of the promoter regions used in this study

P_{CMV} CMV promoter from plasmid vector pcDNA3

acattgattattgactagttattaatagtaatcaattacggggtcattagttcatagcccatatatggagttccgcgttacataacttacggtaaatggcccgcctg
gctgaccgcccaacgacccccgcccattgacgtcaataatgacgtatgttcccatagtaacgccaatagggactttccattgacgtcaatgggtggactatttacgg
taaactgcccacttggcagtacatcaagtgtatcatatgccaagtacgccccctattgacgtcaatgacggtaaatggcccgcctggcattatgcccagtacatgac
cttatgggactttcctacttggcagtacatctacgtattagtcatcgctattaccatggtgatgcggttttggcagtacatcaatgggcgtggatagcggtttgact
cacggggatttccaagtctccaccccattgacgtcaatgggagtttgttttggcaccaaaatcaacgggactttccaaaatgtcgtaacaactccgccccattgacg
caaatgggcggtaggcgtgtacggtgggaggtctatataagcagagctc

P_{MIN} Minimal promoter

tagagggtatataatggaagctcgacttccag

U6 promoter

gatccgacgcgccatctctaggcccgcgccggccccctcgcacagacttgtgggagaagctc
ggctactcccctgccccggttaatttgcatataatatttcctagtaactatagaggcttaatgtgcgataaaagacagataatctgttcttt
ttaatactagctacattttacatgataggcttggatttctataacttcgtatagcatacattatacgaagttataaacagcacaaaaggaaa
ctcaccctaactgtaaagtaattgtgtgttttgagactataagtatcccttggagaaccaccttgttgg

7x gRNA binding site [b] tcttccgtttccacatct

ccgtcttccgtttccacatcttcacggataccaaggtggcaccggtttgattgcacagcccgtcttccgtttccacatcttcacggtgctggcagccggggtaccgcaggca
agtcgcccgtcttccgtttccacatcttcacggatggaatcacggcggccgcccgtatctcatccccgtcttccgtttccacatcttcacggaatgcgtcggcgtgcggccg
ctgccagttgcaccgtcttccgtttccacatcttcacggttggctgtcctagggtacctggacgctccttgccgtcttccgtttccacatcttcacggtggctggtgccgcacc
ggtaagcagacggccgccgtcttccgtttccacatcttcacggggatcctgtacgggccaga

10x gRNA binding site[ab] gcttcttccgtttccacatct

Cggtggcaccggtttgattgcacagctttactgctgctcccgcttcttccgtttccacatcttgctggcagccggggtaccgcaggcaagtcgctttactgctgctcccgctt
cttccgtttccacatctatggaatcacggcggccgcccgtatctcatcctttactgctgctcccgcttcttccgtttccacatctaatgcgtcggcgtgcggccgctgccagttg
catttactgctgctcccgcttcttccgtttccacatcttggctgtcctagggtacctggacgctccttgtttactgctgctcccgcttcttccgtttccacatcttggctggtgccg
caccggtaagcagaactagaggtggcaccggtttgattgcacagctttactgctgctcccgcttcttccgtttccacatcttgctggcagccggggtaccgcaggcaagtc
gctttactgctgctcccgcttcttccgtttccacatctatggaatcacggcggccgcccgtatctcatcctttactgctgctcccgcttcttccgtttccacatctaatgcgtcggc
gtgcggccgctgccagttgcatttactgctgctcccgcttcttccgtttccacatcttggctgtcctagggtacctggacgctccttgtttactgctgctcccgcttcttccgtttc
cacatct

Supplementary Table 4. The amount of transfected plasmids in each well of 96-well plate used for realization of individual logic functions using the SEAP activity readout

Microscopy, Figure 1c, Supplementary figure 1, 2a, b

Input plasmid	ng	Control	ng
TagRFP_FURS_TD_TEVs_KKYL	70	tagBFP	50
TagRFP_FURs_TD_TEVs_KKMP	70	tagBFP	50
TagRFP_TEVs_KDEL	70	tagBFP	50
TagRFP_FURS_TD_TEVs_KKYL	70	ER-CFP	80
TagRFP_FURs_TD_TEVs_KKMP	70	ER-CFP	80
TagRFP_TEVs_KDEL	70	ER-CFP	80
TagRFP_FURS_TD_TEVs_KKYL	70	GFP-ST<br>KRΦ-BFP	80<br>100
TagRFP_FURs_TD_TEVs_KKMP	70	GFP-ST<br>KRΦ-BFP	80<br>100
TagRFP_TEVs_KDEL	70	GFP-ST<br>KRΦ-BFP	80<br>100
TagRFP_TD_TEVs	70	tagBFP	50

Westerm blot, Supplementary figure 2 c, d

Input plasmid	ng
SEAP-TEVs-KDEL	1500
SEAP-3xFURs-TD-TEVs-KKYL	1500

membER and lumER secretion, Figure 1d, e, Supplementary figure 3

Input protease	ng	Reporter plasmid	ng
erTEVP	0<br>10	SEAP_TEVs_KDEL	50
erSbMVp	0<br>10	SEAP_SbMVs_KDEL	50
TEVp	0<br>80	SEAP_3xFURs_TM_3xTEVs_KKYL	60
PPVp	0<br>80	SEAP_3xFURs_TM_3xPPVs_KKYL	60
SbMVp	0<br>80	SEAP_3xFURs_TM_3xSbMVs_KKYL	60
SuMMVp	0<br>80	SEAP_3xFURs_TM_3xSuMMVs_KKYL	60
TEVp	0<br>80	SEAP_3xFURs_TM_3xTEVs_KKMP	60
TEVp	0<br>20<br>40<br>80	SEAP_3xFURs_TM_3xTEVs_KKMP	60
erTEVp	0<br>0.4<br>1.2<br>5<br>10	SEAP_3xFURs_TM_3xTEVs_KKYL	60
erTEVp	0<br>10<br>20<br>40<br>80	Gluc _TEVs_KDEL	60
TEVp<br>FURp	0<br>60<br>0<br>10<br>20<br>40<br>80	SEAP_3xFURs_TM_3xTEVs_KKYL	60
erPPVp	0<br>5<br>10<br>20	SEAP_PPVs_KDEL	50
erPPVp (N23Q, T173G)	0<br>5<br>10<br>20	SEAP_PPVs_KDEL	50
TEVp	0<br>20<br>40<br>80	SEAP_3xFURs_TM_1xTEVs_KKYL	30
TEVp	0<br>20<br>40<br>80	SEAP_3xFURs_TM_3xTEVs_KKYL	30

membER and lumER inducible secretion, Figure 2b, Supplementary figure 4, 5

Input plasmids	ng	Reporter plasmid	ng	Inducer molecule
FKBP_cerTEVp:<br>FRB_nerTEVp	20:20<br>40:40<br>60:60	SEAP_TEVs_KDEL	90	rapamycin
FKBP_cTEVp:<br>FRB_nTEVp	20:20<br>40:40<br>60:60	SEAP_3xFURs_TM_3xTEVs_KKYL	90	rapamycin
ABI_cTEVp:<br>PYL1_nTEVp	20:20<br>40:40<br>60:60	SEAP_3xFURs_TM_3xTEVs_KKYL	90	ABA
FKBP_cTEVp:<br>FRB_nTEVp	20:20<br>40:40<br>60:60	SEAP_3xFURs_TM_3xTEVs_KKMP	90	rapamycin
FKBP_cerTEVp:<br>FRB_nerTEVp	20:20<br>40:40<br>60:60<br>80:80	SEAP_TEVs_KDEL	20<br>40<br>60<br>80	rapamycin
ABI_cerTEVp<br>PYL1_nerTEVp	10:10<br>20:20<br>40:40	SEAP_TEVs_KDEL	20<br>40<br>60<br>80	ABA

Secretion kinetics, Figure 2c, d, Supplementary figure 6, 7

Input plasmids	ng	Reporter plasmid	ng	Inducer molecule
FKBP_cerTEVp:<br>FRB_nerTEVp	20:20	SEAP_TEVs_KDEL	60	rapamycin
FKBP_cTEVp:<br>FRB_nTEVp	80:80	SEAP_3xFURs_TM_3xTEVs_KKYL	60	rapamycin
ABI_cTEVp:<br>PYL1_nTEVp	80:80	SEAP_3xFURs_TM_3xTEVs_KKYL	60	ABA
dCas9:FKBP<br>FRB:VPR<br>sgRNA [b]	25<br>25<br>25	pMIN_SEAP	60	rapamycin
dCas9:ABI<br>PYL1:VPR<br>sgRNA [b]	25<br>25<br>25	pMIN_SEAP	60	ABA

Addition of KDELR and FURp, Supplementary figure 3f, 8

Input plasmids	ng	Reporter plasmid	ng
TEVp<br>FURp		SEAP_3xFURs_TM_3xTEVs_KKYL	1000
erTEVp<br>KDELR		SEAP_TEVs_KDEL	1000

Secretion with added Eeyarestatin I, Supplementary figure 9

Input plasmids	ng	Reporter plasmid	ng	Inducer molecule
FKBP_cerTEVp<br>FRB_nerTEVp	150<br>150	SEAP_TEVs_KDEL	1000	rapamycin
FKBP_cTEVp<br>FRB_nTEVp<br>FURp	600<br>600<br>100	SEAP_3xFURs_TM_3xTEVs_KKYL	1000	rapamycin

Microscopy – lumER secretion, Figure 3a,b, Supplementary figure 10

Input plasmid	ng	Protease	ng	Transfection control	ng
GFP11x7-Fas-Env<br>SS-GFP1-10-TEVs-KDEL	150<br>150	erTEVp	30	iRFP	20
GFP11x7-Fas-Env<br>SS-GFP1-10-TEVs-KDEL	150<br>150	FKBP_cerTEVp:<br>FRB_nerTEVp	30<br>30	iRFP	20

Microscopy – membER secretion, Figure 3c,d, Supplementary figure 11					
Input plasmid	ng	Protease	ng	Transfection control	ng
SS-GFP1-10-FURs-KD-TEVs-KKYL	100	FKBP_cTEVp:	100	iRFP	20
GFP11x7-Fas-Env-iRFP	100	FRB_nTEVp	100		
SS-GFP1-10-FURs-KD-TEVs-KKYL	100	TEVp	100	iRFP	20
GFP11x7-Fas-Env-iRFP	100		100		

membER and lumER orthogonality, Figure 4				
Input plasmids	ng	Reporter plasmid	ng	Inducer molecule
erTEVp	10	Bchain_Myc_Gluc_Achain_TEVs_KDEL :	15	/
TEVp	80	SEAP_FURs_TD_TEVs_KKYL	30	
FKBP_cerTEVp : FRB_nerTEVp	10:10	Bchain_Myc_Gluc_Achain_TEVs_KDEL :	15	ABA/rapamycin
ABI_cTEVp: PYL1_nTEVp	80:80	SEAP_FURs_TD_TEVs_KKYL	30	

Logical processing, Figure 5, Supplementary figure 13			
Input plasmids	ng	Reporter plasmid	ng
cTEV*-AP4-SbMVs-P3-nTEV	10	SEAP_3xFURs_TD_TEVs_KKYL	30
P4-PPVs-cTEV	5		
PPVp	90		
SbMVp	60		
cTEV*-AP4-SbMVs-P3-nTEV	10	SEAP_FURs_TD_TEVs_KKYL	30
P4-PPVs-cTEV	5		
SbMVp	60		
PPVp	40		
P3-TEVs-KDEL	20	SEAP-P4	10
erTEVp	5		
erSbMVp	5	SEAP-P4-SbMVs-KDEL	10
P3-TEVs-KDEL	40	SEAP-P4-SbMVs-KDEL	10
erTEVP	5		
erSbMVp	5		

OFF switch, Figure 5d, , Supplementary figure 14					
Input plasmid	ng	Protease	ng	Protease	ng
SEAP_3xFURs_TD_TEVs_KKYL	20	FKBP_PPVs_cTEVp<br>FRB_PPVs_nTEVp	30<br>30	PPVp	0<br>20<br>40<br>80<br>120
		TEVp	30		
SEAP_3xFURs_TD_TEVs_KKYL<br>furin	300<br>100	FKBP_PPVs_cTEVp<br>FRB_PPVs_nTEVp	300<br>300	ABI_cPPVp<br>PYL_nPPVp	800<br>800

ER-stress and cell viability , Supplementary figure 15			
Input plasmids	ng		
Preproinsulin_TEVs_KDEL (lumER-insulin)	500	FKBP_cerTEVp:<br>FRB_nerTEVp	25<br>25
Preproinsulin_TEVs_KKYL (membER-insulin)	500		
CAD-insulin	500		
Preproinsulin_TEVs_KDEL (lumER-insulin)	50<br>100<br>200	FKBP_cerTEVp:<br>FRB_nerTEVp	5<br>5
Preproinsulin_TEVs_KKYL (membER-insulin)	50<br>100<br>200		
CAD-insulin	50<br>100<br>200		

Insulin and IL10 secretion, Figure 6, Supplementary figure 16				
Input plasmids	ng	Input plasmids	ng	Inducer molecule
FKBP_cerTEVp:<br>FRB_nerTEVp	70<br>70	Preproinsulin_TEVs_KDEL	550	rapamycin
dCas9:ABI<br>PYL1:VPR<br>sgRNA	150<br>150<br>150	pMIN_preproinsulin	450	ABA
erTEVp	0<br>10<br>20<br>50<br>100	Bchain_Gluc_Achain_TEVs_KDEL	20	/
FKBP_cerTEVp:<br>FRB_nerTEVp	0:0<br>5:5<br>10:10<br>20:20<br>30:30	Bchain_Gluc_Achain_TEVs_KDEL	20	rapamycin
FKBP_cerTEVp:<br>FRB_nerTEVp	5:5	hIL10-TEVs-KDEL	50	rapamycin

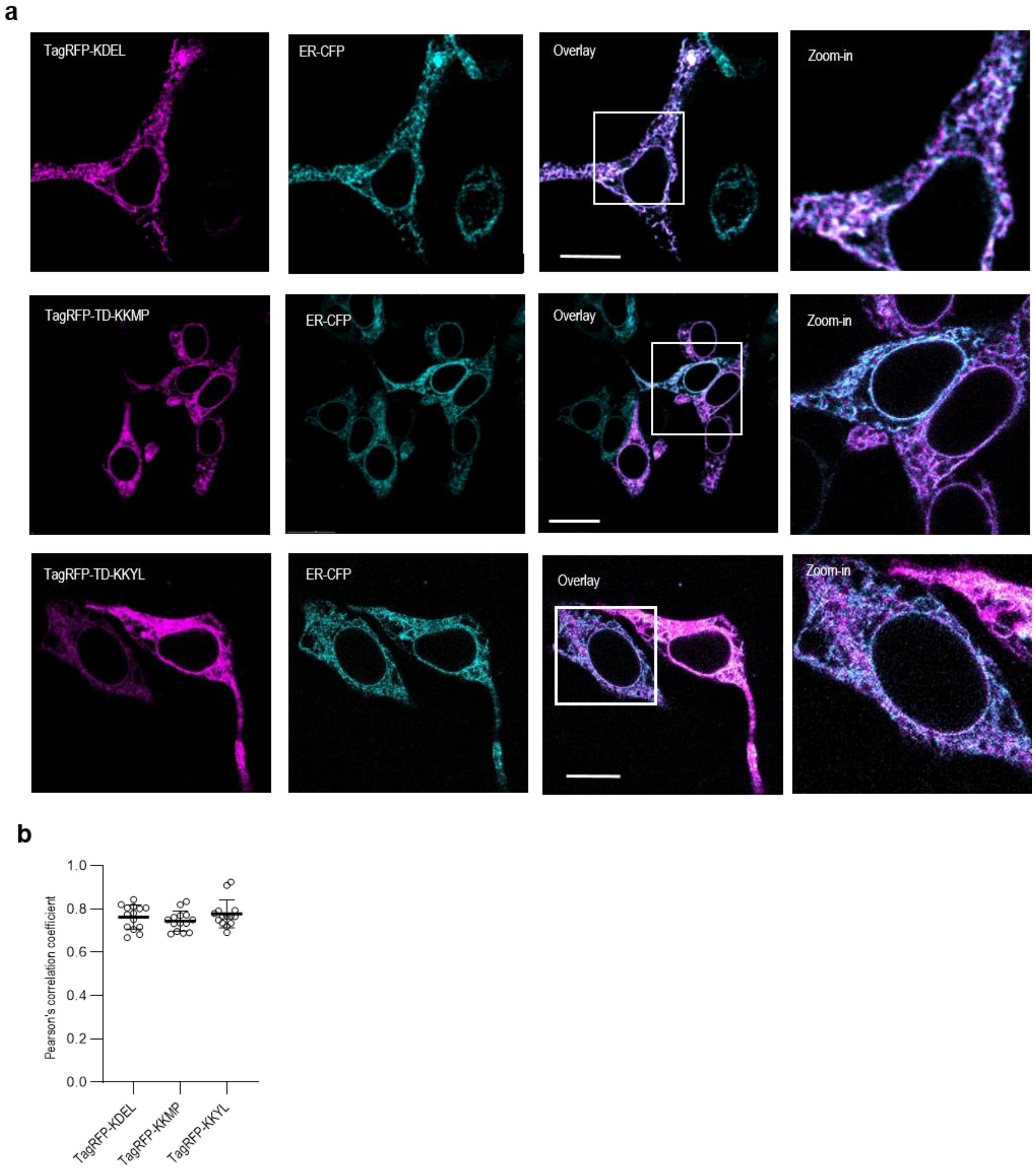

Supplementary figure 1: Cellular localization of lumER and membER constructs. a, TagRFP-KDEL, TagRFP-TD-KKMP and TagRFP-TD-KKYL were coexpressed with an ER marker (ER-CFP)[1]. Images are representative of 3 independent experiments. **b**, Colocalization analysis from (**a**) shown as scatter plot (mean + s.d.). Pearson's correlation coefficient was calculated for TagRFP and CFP (ER marker) (n = 10 images). Values are representative of 3 independent experiments. Source data are provided as a Source Data file.

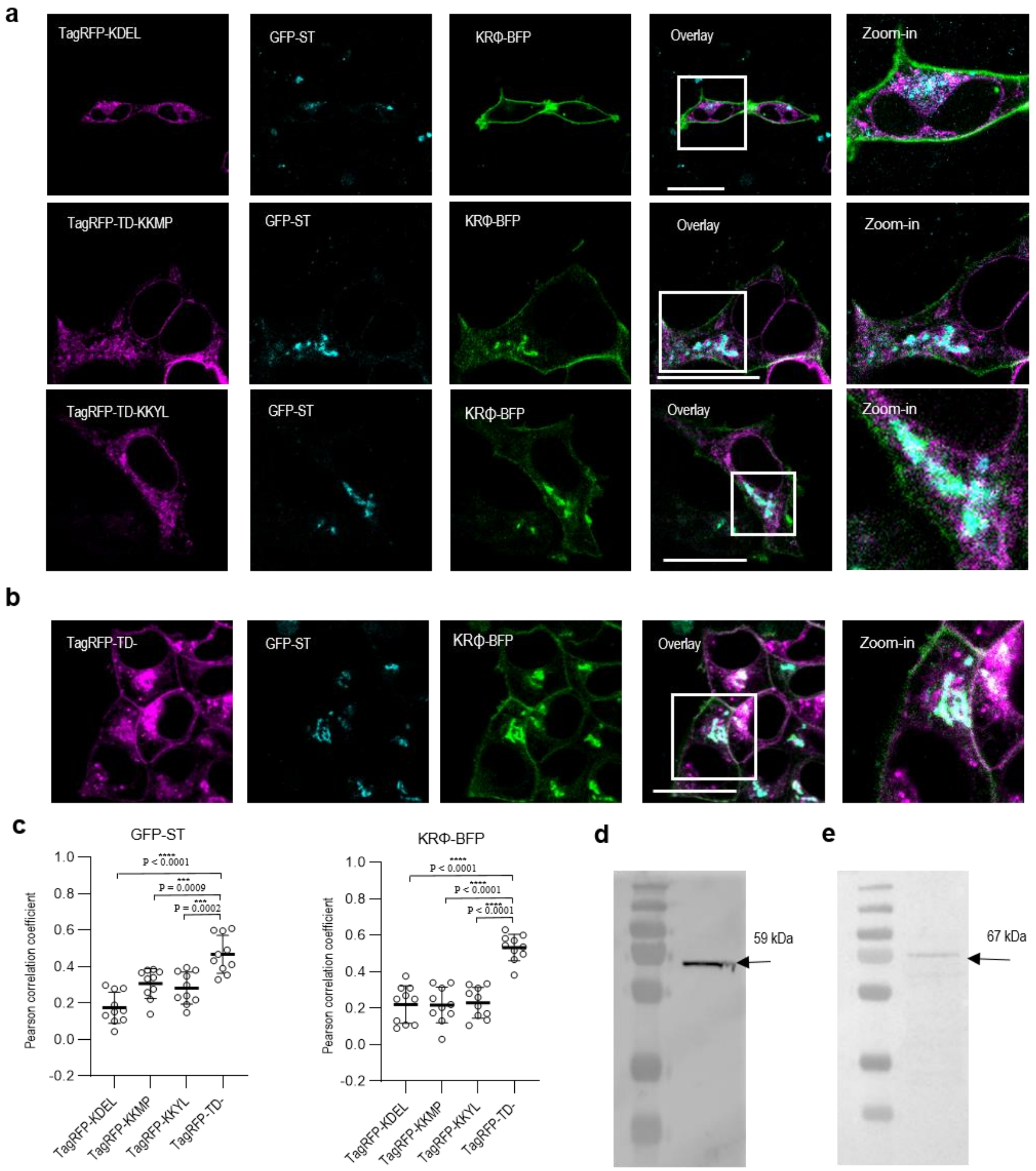

Supplementary figure 2: Expression pattern of lumER and membER constructs. a, TagRFP-KDEL, TagRFP-TD-KKMP, TagRFP-TD-KKYL and **b**, TagRFP-TD-TEVs, lacking a C-terminal retention signal were coexpressed with the GFP– sialyltransferase (GFP-ST) fusion protein, which localizes in the GA[2], and KRΦ-BFP, which anchors the plasma membrane[3]. The addition of the retention signal localizes the protein in the ER, preventing its migration to the GA and plasma membrane. In contrast to constructs featuring a retention sequence, TagRFP signal is detected also at the plasma membrane, indicating that the protein without a retention signal trafficked to the cell surface. Scale bar = 20 µm. Images are representative of 3 independent experiments. **c**, Colocalization analysis from (**a** and **b**) shown as scatter plot (mean + s.d.). Pearson's correlation coefficient was calculated for TagRFP and GFP (GA marker) or BFP (plasma membrane marker) (n = 10 images). Significance was

tested by one-way analysis of variance (ANOVA) with Dunnett's comparison. $*P < 0.05$, $**P < 0.01$, $***P < 0.001$, $****P < 0.0001$. Values are representative of two independent experiments. Plasmids coding either SEAP-TEVs-KDEL (AU1 tag) (**d**) or SEAP-FURs-TD-TEVs-KKYL (AU1 tag) (**e**) were transfected into HEK293T, lysed after 48h and their expression was verified by Western blot. A band at 59 kDa corresponds to SEAP-TEVs-KDEL and a band at 67 kDa corresponds to SEAP-FURs-TD-TEVs-KKYL. Source data are provided as a Source Data file.

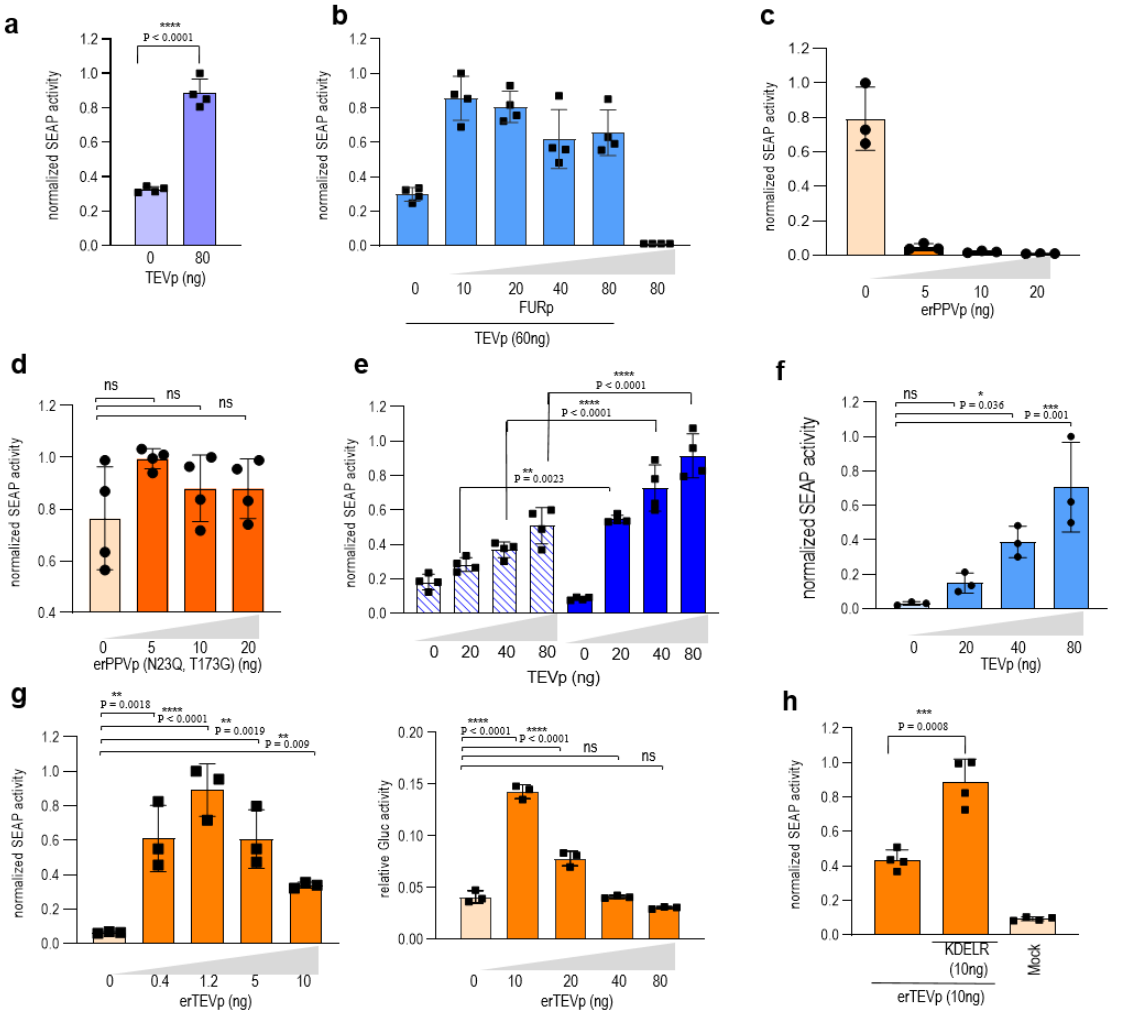

Supplementary figure 3: Proteolysis-based protein secretion. a, Effect of a TEVp-cleavable C-terminal KKMP ER retention sequence. **b**, Co-transfection of furin protease (FURp) increases the amount of secreted SEAP in the membER system. **c**, Regulation of protein secretion by the erPPVp targeting SEAP-PPVs-KDEL. The protease lacks the catalytic activity inside ER. **d**, Regulation of protein secretion of a mutated version of erPPVp(N23Q, T173G) targeting SEAP-PPVs-KDEL. **e**, In combination with the membER system, the addition of three TEVs between the transmembrane domain and retention signal (Dark blue) increased the release of SEAP after the addition of TEVp, when compared with a single TEVs (stripe pattern). **f**, TEVp concentration dependence on protein secretion with the membER system, detected by the secreted SEAP. **g**, erTEVp concentration dependence on protein secretion with the lumER system, detected by the secreted SEAP (left) and Gaussia luciferase (Gluc, right). **h**, Cotransfection of KDELR1 increases the retention capacity of the system. Values are the mean of three or four cell cultures ± s.d. and are representative of two independent experiments. Significance was tested by a one-way analysis of variance (ANOVA) with Dunnett's comparison. *P < 0.05, **P < 0.01, ***P < 0.001, ****P < 0.0001. Source data are provided as a Source Data file.

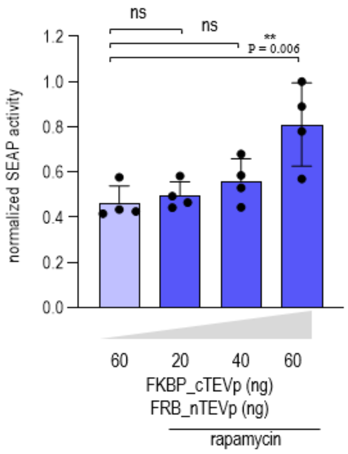

Supplementary figure 4: Rapamycin inducible secretion of the membER system with a KKMP retention signal. Values are the mean of four cell cultures ± s.d. and are representative of two independent experiments. Significance was tested by a one-way analysis of variance (ANOVA) with Dunnett's comparison. **P = 0.006. Source data are provided as a Source Data file.

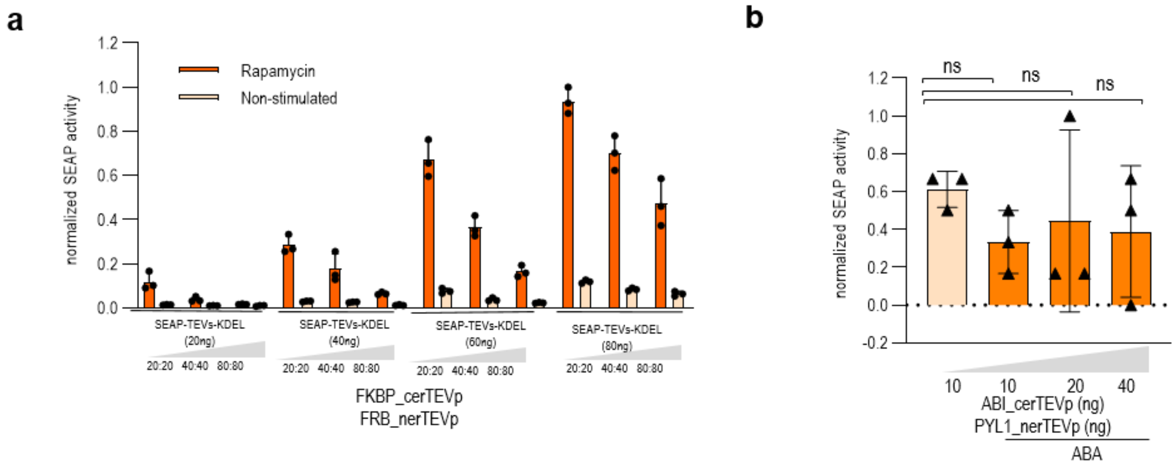

Supplementary figure 5: Inducible secretion of SEAP by CID with the lumER system. a, 20, 40 or 80 ng of pcDNA3.1 comprising P_{CMV}_FKBP-cerTEVp and P_{CMV}_FRB-nerTEVp (er-rapa-TEV) were co-transfected with 20, 40, 60 or 90ng of pcDNA P_{CMV}_SEAP-TEVs-KDEL. SEAP activity in the medium was measured 16h after stimulation with rapamycin. Increasing the amount of the split protease decreases the fold difference in secreted SEAP between stimulated and non-stimulated cells. **b**, Regulation of proteins secretion by ABI-cerTEV/PYL1-nerTEVp and SEAP-TEVs-KDEL. The dimerization potential of ABI/PYL1 domains is diminished inside of ER (er-aba-TEV). Values are the mean of three or four cell cultures ± s.d. and are representative of two independent experiments. Significance was tested by a one-way analysis of variance (ANOVA) with Dunnett's comparison. Source data are provided as a Source Data file.

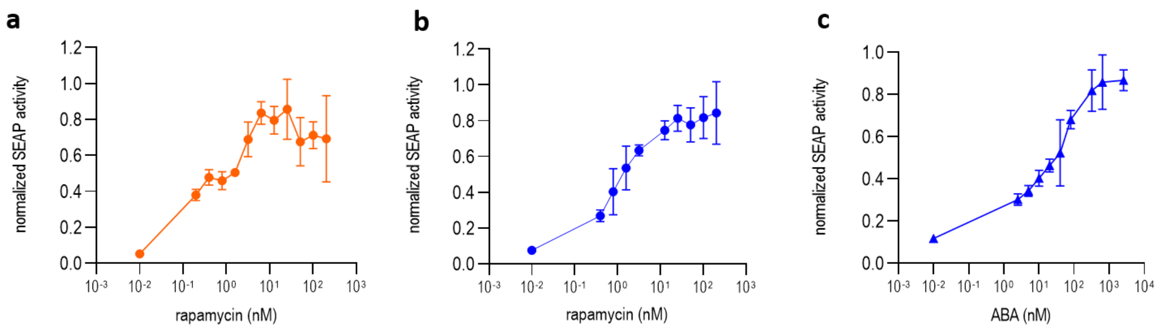

Supplementary figure 6: SEAP secretion dependence on the concentration of chemical inducer. a, Titration of rapamycin on SEAP-TEVs-KDEL (lumER system). **b**, Titration of rapamycin on SEAP-FURs-TM-TEVs-KKYL (membER system). **c**, Titration of ABA on SEAP-FURs-TM-TEVs-KKYL (membER system). Values are the mean of three cell cultures ± s.d. and are representative of two independent experiments. Source data are provided as a Source Data file.

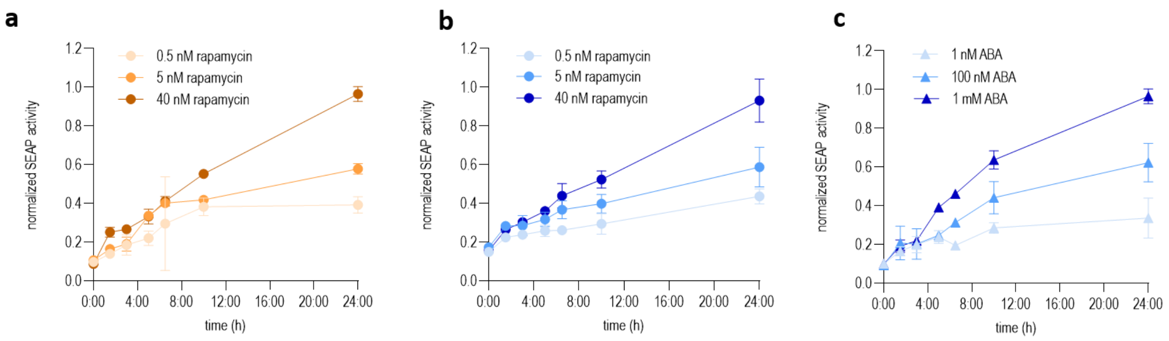

Supplementary figure 7: Secretion kinetics of lumER and membER with varying concentration of chemical inducer. HEK293T cells were transfected with either the lumER (orange, panel **a**) or membER (blue, panels **b** and **c**) system in combination with an inducible split TEVp. Two days after transfection cells were stimulated with rapamycin (round symbols) at a final concentration of 0.5 nM, 5nM and 40 nM, or ABA (triangle symbols) at a final concentration of 1 nM, 100 nM and 1 mM. Media was sampled at specific time points and SEAP activity was measured. Values are the mean of three cell cultures ± s.d. and are representative of two independent experiments. Source data are provided as a Source Data file.

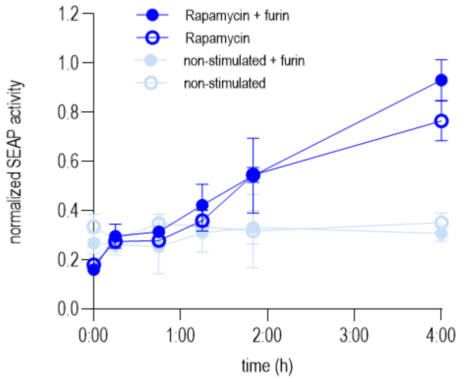

Supplementary figure 8: The effect of furin protease on the kinetics of SEAP secretion with the membER system. Co-transfection of FURp did not significantly impact the secretion kinetics in the first 4h after induction of secretion. Values are the mean of four cell cultures ± s.d and are representative of two independent experiments. Source data are provided as a Source Data file.

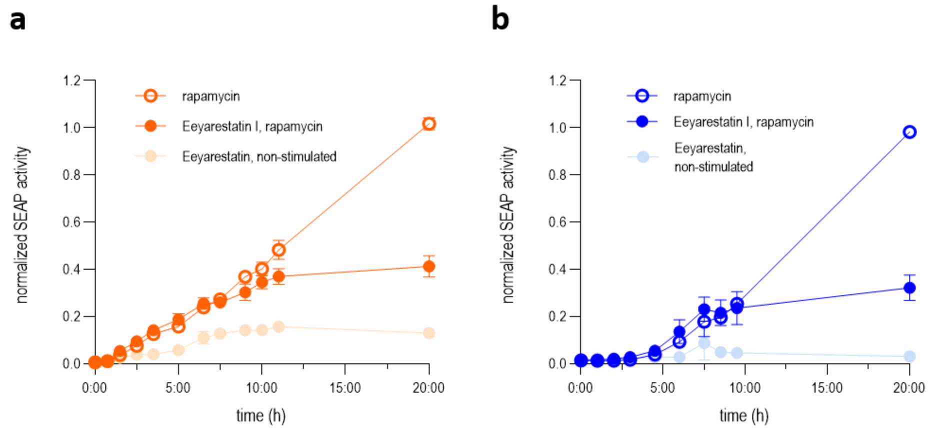

Supplementary figure 9: Retention capacity of secretion systems. Cells were treated with an inhibitor of ER transport, Eeyarestatin I. After stimulation with rapamycin, cell medium was harvested at specified time points and SEAP activity was measured. Both the lumER (**a**) and membER (**b**) system release most of the stored protein around 10h after induction of secretion. The remaining secretion in cells not treated with Eeyarestatin I is most likely due to the re-storing of ER with newly synthesized protein. Values are the mean of three cell cultures ± s.d. and are representative of two independent experiments. Source data are provided as a Source Data file.

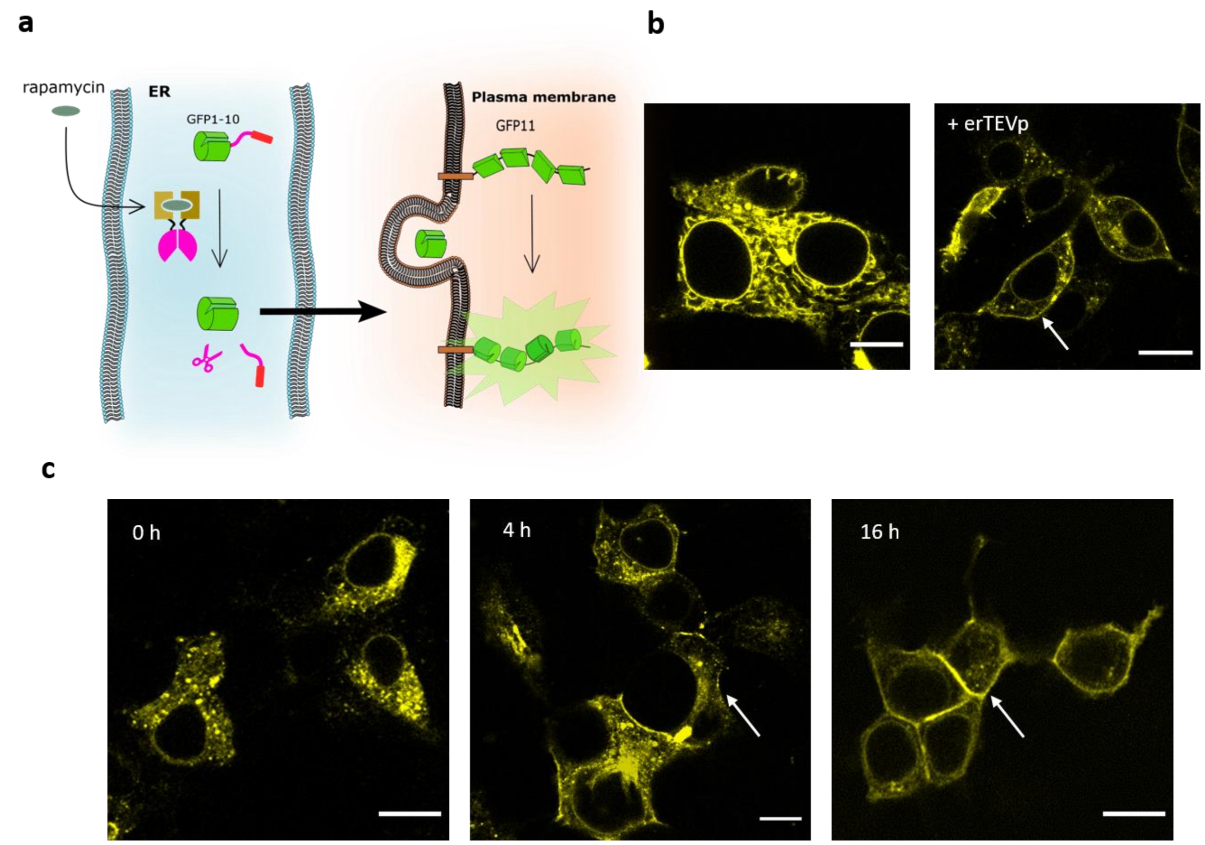

Supplementary figure 10: Visualization of protein trafficking in the lumER system. A split GFP fragment, composed of the 10 β-strands of the fluorescent protein (GFP1-10), was fused to the lumER system, while the complementary fragment, composed of 7 repeats of the 11th β-strand (GFP11x7), was fused to the FAS receptor transmembrane domain, localizing it to the plasma membrane. Removal of the retention signal allows GFP1-10 to move to the membrane and associate with its complementary part, reconstituting full GFP, which can be detected under the microscope. **b**, GFP fluorescence was measured before and after co-transfection of erTEVp. After the addition of the protease, the signal was detected on the cell membrane (white arrow), where it was previously undetected. **c**, FKBP-cerTEVp /FRB-nerTEVp was used inducible reconstitution of the split protease and the removal of the retention signal. GFP fluorescence was visualized before stimulation of cells with rapamycin, 4h after and 16h after the addition of rapamycin. Scale bar = 10 μm. Images are representative of three independent experiments.

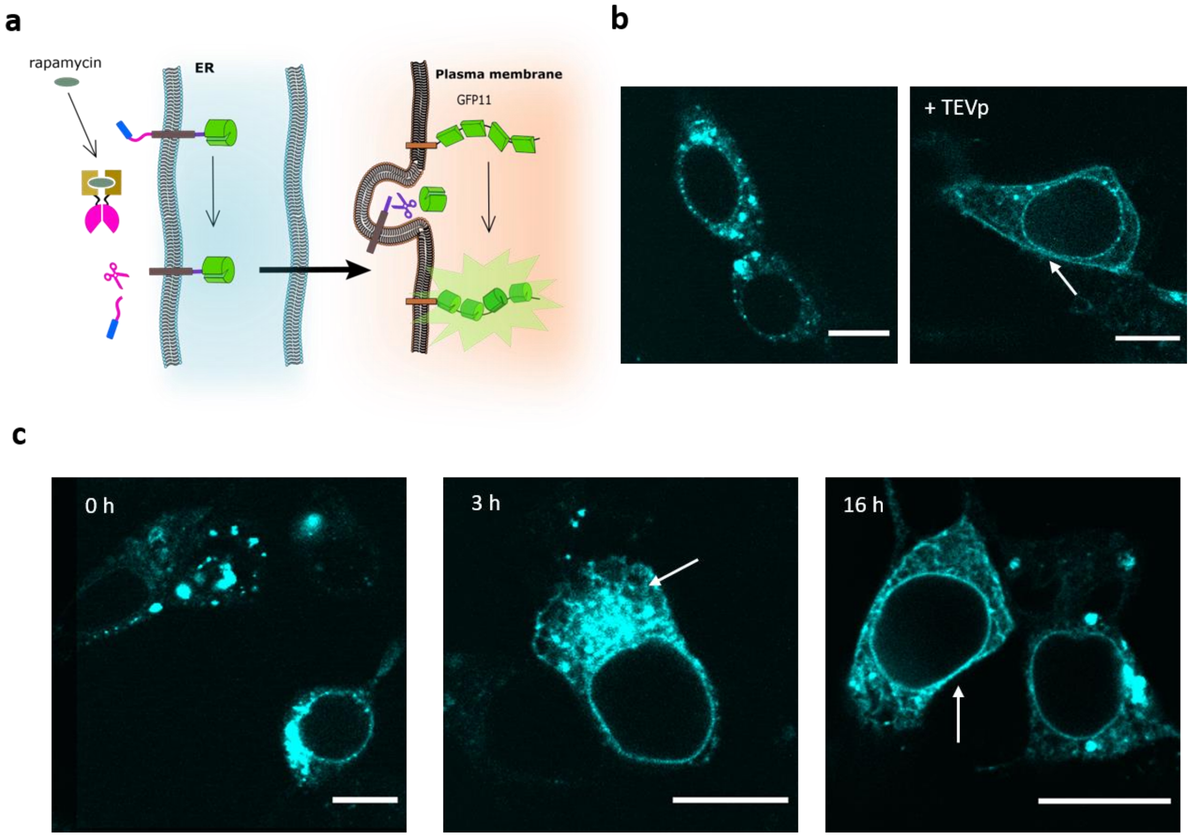

Supplementary figure 11: Visualization of protein trafficking in the membER system. Similar as the lumER system, a GFP1-10 was fused to the membER system, while the complementary fragment, GFP11x7, was fused to the FAS receptor transmembrane domain. A cytosolic protease facilitates the removal of the KKYL signal, allowing GFP1-10 to move to its complementary fragment, thus regaining its fluorescent activity. **b**, GFP fluorescence was measured before and after co-transfection of TEVp. After the addition of the protease, the signal was detected on the cell membrane (white arrow), where it was previously undetected. **c**, FKBP-cTEVp /FRB-nTEVp was used inducible reconstitution of the split protease and the removal of the retention signal. GFP fluorescence was visualized before stimulation of cells with rapamycin, 3h after and 16h after the addition of rapamycin. Scale bar = 10 µm. Images are representative of three independent experiments.

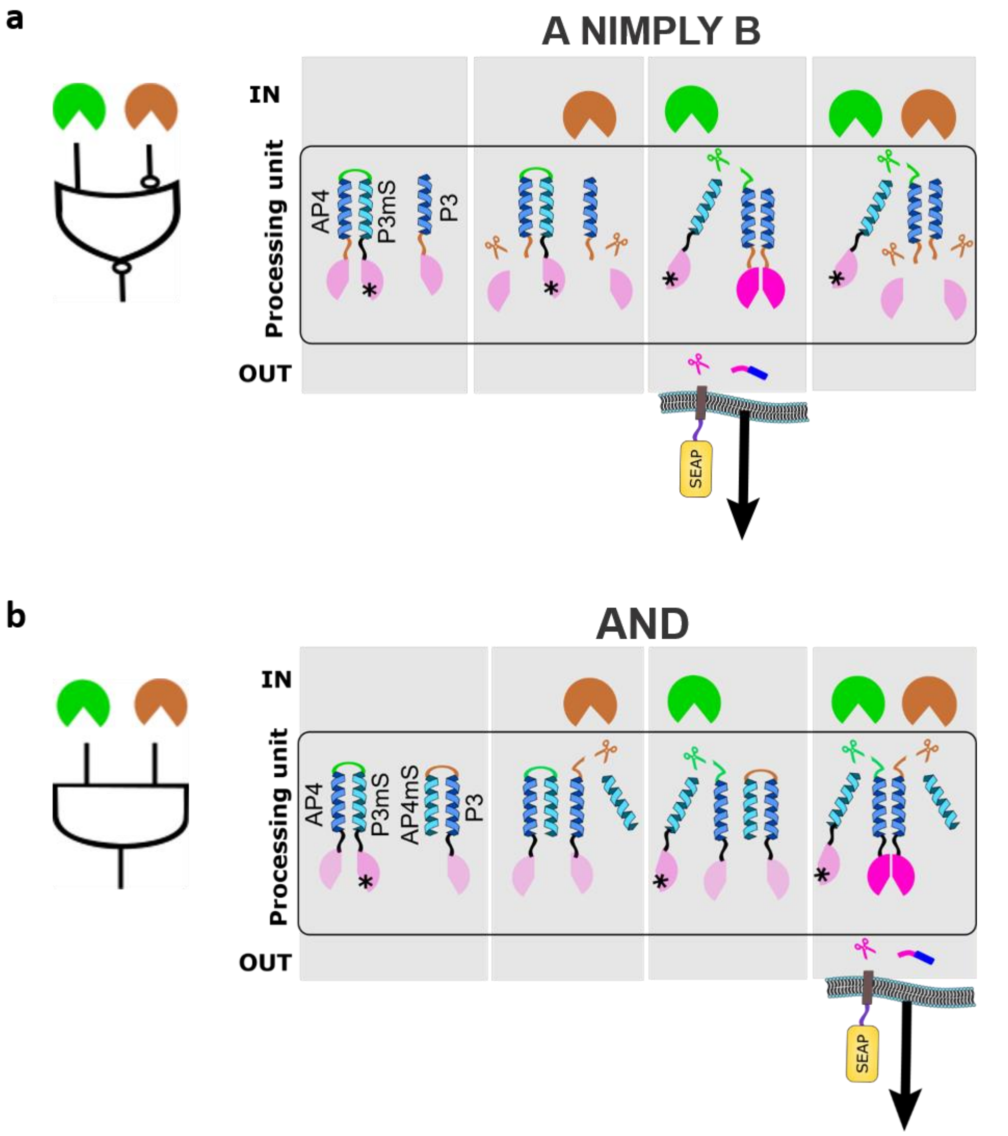

Supplementary figure 12: Schematic representation of the constructs used for A nimply B and AND Boolean SPOC logic circuits used to regulate membER-SEAP secretion . a, design of A nimply B logical function. The constructs used for signal processing were nTEV-SbMVs-AP4-PPVs-P3mS-cTEV* and P3-SbMVs-cTEV. An output in the form of a reconstituted TEVp, which further regulates the secretion of SEAP, is produced only when PPVp is present, but not SbMVp. This is achieved through the introduction of an inhibitory coil P3mS, which prevents the reconstitution of the two protease fragments through the interaction of the complementary P3/AP4 coiled-coils. An introduction of an inactive cTEV fragment (indicated with cTEV*) further prevents unwanted coupling of the split protease fragments. The removal of the inhibitory P3mS-cTEV* is achieved by the cleaving of the PPVs (green line), which is positioned in the linker connecting the AP4/P3mS coiled coils. A negative regulation with SbMVp is achieved by positioning a SbMVs (brown line) between the active fragments of the split TEVp and its adjacent coil. **b**, design of AND logical function. The constructs used for AND signal processing were nTEV-AP4-PPVs-P3mS-cTEV* and AP4mS-SbMVs-P3-nTEV. In this setting both PPVp and SbMVp need to be present in order to remove the P3mS-cTEV* and AP4mS inhibitory coils and allow for the coupling of the split TEVp fragments through P3/AP4 coiled-coil interaction.

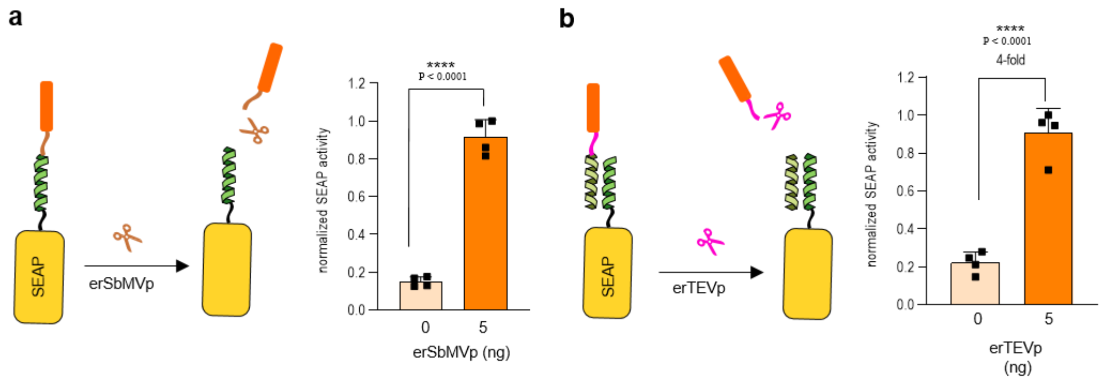

Supplementary figure 13: Retention of SEAP inside the ER through coiled-coil interactions. a, SEAP was fused to a P4 coiled-coil forming peptide and a KDEL retention signal, separated by SbMVs. Upon the addition of erSbMVp, secretion is induced. **b**, Addition of P3-TEVs-KDEL significantly decreases the secretion of SEAP-P4, which itself does not contain a retention signal. Upon the addition of erTEVp, SEAP is detected in the media. Values are the mean of four cell cultures ± s.d. and are representative of two independent experiments. An unpaired two-tailed t test (after equal variance was assessed with the F test assuming normal data distribution) was used for the statistical comparison of the data. **** P < 0.0001. Source data are provided as a Source Data file.

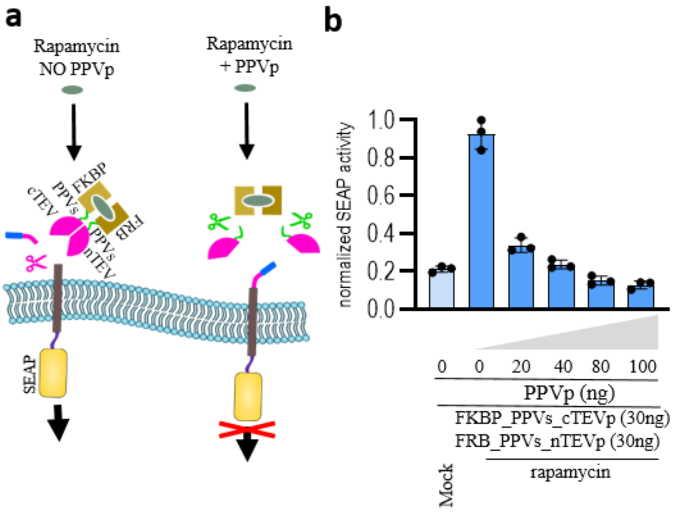

Supplementary figure 14: A simplified A nimply B logical function for the membER system. a, A PPVp cleavage site (PPVs) was inserted between the FKBP/FRB dimerization domains and TEV protease fragments. The reconstitution of TEVp can be inhibited by the addition of PPVp, **b**, Cells were stimulated with rapamycin to induce secretion. Cells co-expressing PPVp showed decreased secretion of SEAP, compared to cells where no PPVp was added. Values are the mean of three cell cultures ± s.d. and are representative of two independent experiments. Source data are provided as a Source Data file.

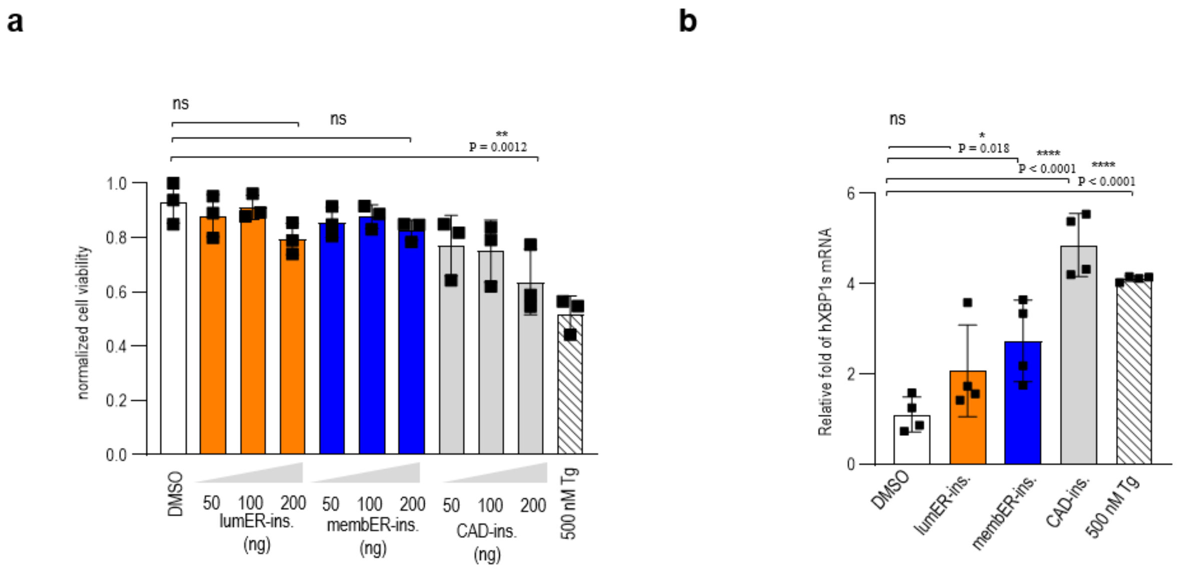

Supplementary figure 15: Cell viability and ER-stress induction of the lumER and membER constructs. a, Cell viability was measured with an ATP determination assay. Cells were transfected with varying amounts of lumER-insulin and membER-insulin and compared with a conditional aggregation domain (CAD)-based system for protein retention inside the ER[4]. Even when overexpressed, the system did not decrease cell viability, whereas overexpression CAD-insulin led to the decrease of cell viability. 500 nM Thapsigargin (Tg) was used to induce ER stress (stripe pattern). **b,** Relative expression of spliced XBP1 mRNA (XBP1s) was measured to determine the unfolded protein response (UPR) under conditions of ER stress[5]. When overexpressed, the membER-insulin lead to a slight increase, while lumER-insulin did not affect XBP1s expression. CAD-insulin expression lead to an increase in XBP1s expression, comparable to that induced by 500 nM Tg. Values are the mean of three and four cell cultures ± s.d. and are representative of two independent experiments. Significance was tested by a one-way analysis of variance (ANOVA) with Dunnett's comparison. *P < 0.05, **P < 0.01, ***P < 0.001, ****P < 0.0001. Source data are provided as a Source Data file.

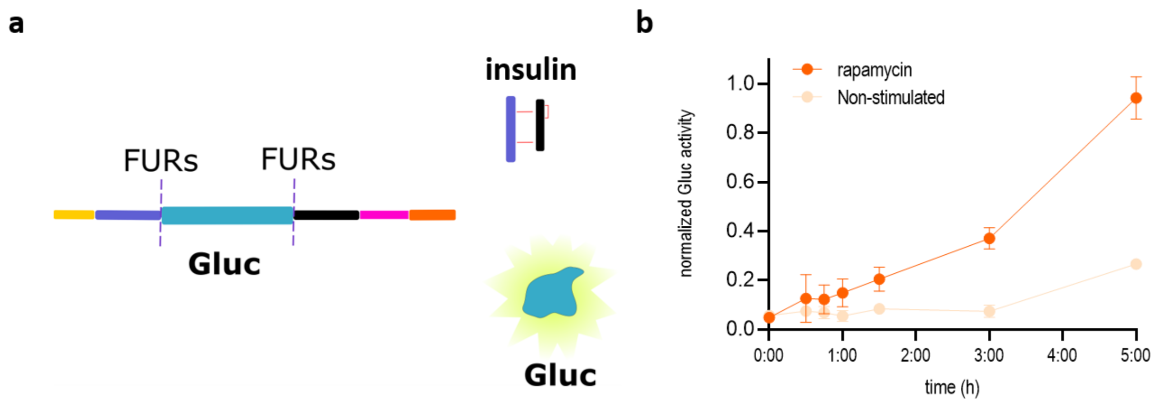

Supplementary figure 16: Monitoring insulin secretion by measuring Gluc activity in the media. As an alternative system to monitor the release of insulin into the cell media after the addition of rapamycin, the C-peptide sequence was replaced by Gaussia luciferase (Gluc) and luciferase activity was measured in the media as a proxy for insulin secretion. Values are the mean of four cell cultures ± s.d. and are representative of two independent experiments. Source data are provided as a Source Data file.

a

b

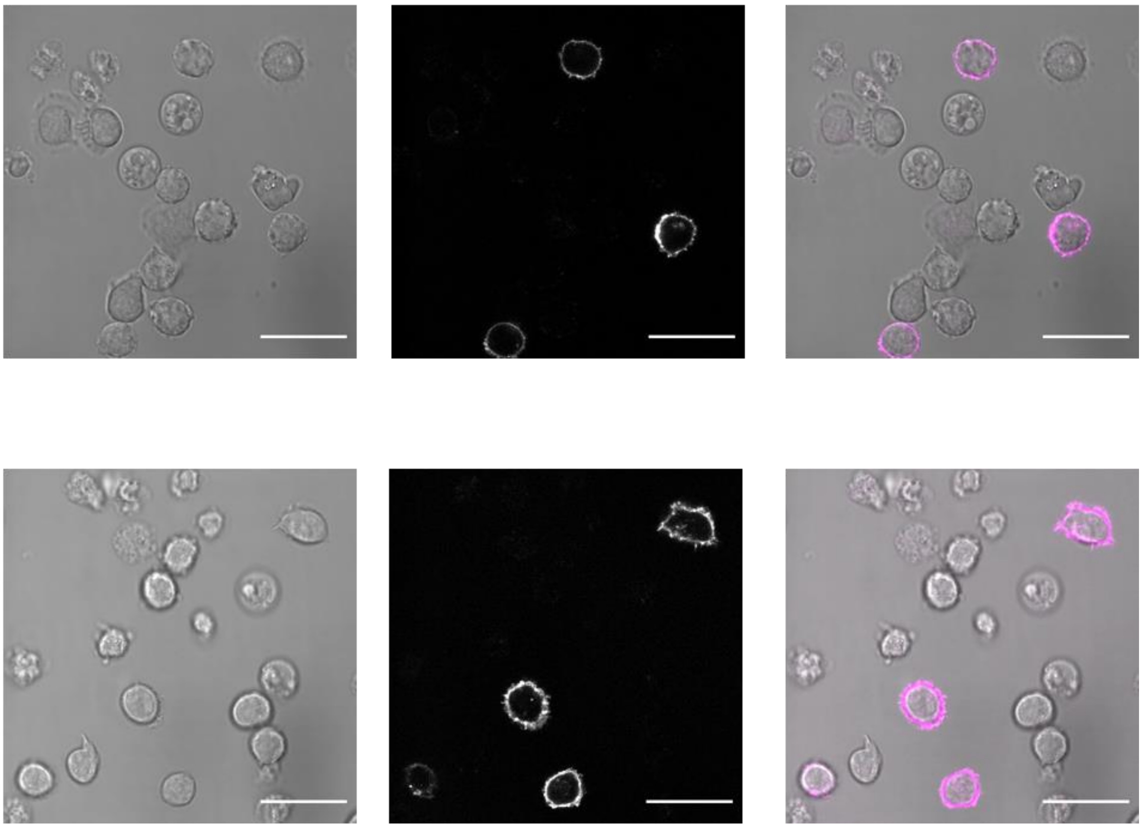

Supplementary figure 17: Imaging of membER CAR-T on cell surface after proteolytic cleavage. a) 24 hours after electroporation, cells were stained with a fluorescent-labeled- antibody (anti-c-Myc 9B11 Alexa Flour 647) to detect translocation of membER CAR T to the plasma membrane. **b)** Electroporation of wtCAR was used as a control. Images are representative of two independent experiments. Scale bar = 25 µm.

References

1. Nilsen, N. J. *et al.* Cellular trafficking of lipoteichoic acid and Toll-like receptor 2 in relation to signaling; role of CD14 and CD36. *J. Leukoc. Biol.* **84**, 280–291 (2008).
2. Schaub, B. E., Berger, B., Berger, E. G. & Rohrer, J. Transition of Galactosyltransferase 1 from Trans -Golgi Cisterna to the Trans -Golgi Network Is Signal Mediated. *Mol. Biol. Cell* **17**, 5153–5162 (2006).
3. Meško, M., Lebar, T., Dekleva, P., Jerala, R. & Benčina, M. Engineering and Rewiring of a Calcium-Dependent Signaling Pathway. *ACS Synth. Biol.* **9**, 2055–2065 (2020).
4. Rivera, V. M. *et al.* Regulation of protein secretion through controlled aggregation in the endoplasmic reticulum. *Science (80-.).* **287**, 826–830 (2000).
5. Hirota, M., Kitagaki, M., Itagaki, H. & Aiba, S. Quantitative measurement of spliced XBP1 mRNA as an indicator of endoplasmic reticulum stress. *Journal of Toxicological Sciences* vol. 31 149–156 (2006).
