# Supplementary material for: Regulation of protein secretion through chemical regulation of endoplasmic reticulum retention signal cleavage
Source: Nat Commun. 2022 Mar 14;13:1323. doi: 10.1038/s41467-022-28971-9 (PMC8904541; doi:10.1038/s41467-022-28971-9)
Supplement: Supplementary file 2 — Reporting Summary [file 41467_2022_28971_MOESM2_ESM.pdf]

nature research

Corresponding author(s): Roman Jerala,

Last updated by author(s): Feb 17, 2022

Reporting Summary

Nature Research wishes to improve the reproducibility of the work that we publish. This form provides structure for consistency and transparency in reporting. For further information on Nature Research policies, see our Editorial Policies and the Editorial Policy Checklist.

Statistics

For all statistical analyses, confirm that the following items are present in the figure legend, table legend, main text, or Methods section.

n/a	Confirmed	
☐	☒	The exact sample size (*n*) for each experimental group/condition, given as a discrete number and unit of measurement
☐	☒	A statement on whether measurements were taken from distinct samples or whether the same sample was measured repeatedly
☐	☒	The statistical test(s) used AND whether they are one- or two-sided<br>*Only common tests should be described solely by name; describe more complex techniques in the Methods section.*
☒	☐	A description of all covariates tested
☒	☐	A description of any assumptions or corrections, such as tests of normality and adjustment for multiple comparisons
☐	☒	A full description of the statistical parameters including central tendency (e.g. means) or other basic estimates (e.g. regression coefficient) AND variation (e.g. standard deviation) or associated estimates of uncertainty (e.g. confidence intervals)
☐	☒	For null hypothesis testing, the test statistic (e.g. *F*, *t*, *r*) with confidence intervals, effect sizes, degrees of freedom and *P* value noted<br>*Give P values as exact values whenever suitable.*
☒	☐	For Bayesian analysis, information on the choice of priors and Markov chain Monte Carlo settings
☒	☐	For hierarchical and complex designs, identification of the appropriate level for tests and full reporting of outcomes
☒	☐	Estimates of effect sizes (e.g. Cohen's *d*, Pearson's *r*), indicating how they were calculated

Our web collection on statistics for biologists contains articles on many of the points above.

Software and code

Policy information about availability of computer code

Data collection	BioTek's Synergy™ Mx Microplate Reader and Gen5 Microplate Data Collection & Analysis software was used to measure absorbance, Orion II Microplate Luminometer from BERTHOLD TECHNOLOGIES and Simplicity 4.2 software was used to measure luminescence, Leica TSC SP5 confocal microscope equipped with an HCX plan apo 963 (NA 1.4) oil-immersion objective was used for cell imaging. Leica LAS AF software was used for image acquisition
Data analysis	GraphPad Prism 8 (http://www.graphpad.com/)

For manuscripts utilizing custom algorithms or software that are central to the research but not yet described in published literature, software must be made available to editors and reviewers. We strongly encourage code deposition in a community repository (e.g. GitHub). See the Nature Research guidelines for submitting code & software for further information.

Data

Policy information about availability of data

All manuscripts must include a data availability statement. This statement should provide the following information, where applicable:

- Accession codes, unique identifiers, or web links for publicly available datasets
- A list of figures that have associated raw data
- A description of any restrictions on data availability

The authors declare that the data supporting the findings of this study are available in the paper and its supplementary information files. The raw data are available from the corresponding author upon reasonable request.

Field-specific reporting

Please select the one below that is the best fit for your research. If you are not sure, read the appropriate sections before making your selection.

☒ Life sciences ☐ Behavioural & social sciences ☐ Ecological, evolutionary & environmental sciences

For a reference copy of the document with all sections, see nature.com/documents/nr-reporting-summary-flat.pdf

Life sciences study design

All studies must disclose on these points even when the disclosure is negative.

Sample size	At least three independent replicates were taken for each data point in an independent experiment. We chose sufficient sample size to establish that the values compared were derived from normal distributions.
Data exclusions	No data were excluded.
Replication	At least two independent experiments were performed with a comparable outcome.
Randomization	No animal or human research participants were used, therefore there was no reason for randomization.
Blinding	No animal or human research participants were used, therefore there was no reason for blinding. Furthermore, all measurements in independent experiment were performed at the same time with the identical settings.

Reporting for specific materials, systems and methods

We require information from authors about some types of materials, experimental systems and methods used in many studies. Here, indicate whether each material, system or method listed is relevant to your study. If you are not sure if a list item applies to your research, read the appropriate section before selecting a response.

Materials & experimental systems

n/a	Involved in the study	
☐	☒	Antibodies
☐	☒	Eukaryotic cell lines
☒	☐	Palaeontology and archaeology
☒	☐	Animals and other organisms
☒	☐	Human research participants
☒	☐	Clinical data
☒	☐	Dual use research of concern

Methods

n/a	Involved in the study	
☒	☐	ChIP-seq
☒	☐	Flow cytometry
☒	☐	MRI-based neuroimaging

Antibodies

Antibodies used	rabbit polyclonal anti-AU1 tag (Abcam ab3401), HRP-conjugated goat anti–rabbit IgG (Abcam ab6721); Myc Tag (9B11) Mouse Ab Alexa Fluor 647 (Cell signaling CY2233S)
Validation	https://antibodyregistry.org/search.php?q=AB_303770<br>https://antibodyregistry.org/search?q=ab6721<br>https://antibodyregistry.org/search.php?q=AB_823474

Eukaryotic cell lines

Policy information about cell lines

Cell line source(s)	HEK293T cells, from ATCC culture collection (ATCC CRL-1126), Jurkat E6.1 cells from ATCC (ATCC TIB-152)
Authentication	provided by ATCC culture collection
Mycoplasma contamination	negative for mycoplasma - guaranteed from the supplier and regularly checked
Commonly misidentified lines (See ICLAC register)	HEK293T and Jurkat E6.1 are not among the misidentified lines
